# High-resolution acoustophoretic 3D cell patterning to construct functional collateral cylindroids for ischemia therapy

**DOI:** 10.1038/s41467-018-07823-5

**Published:** 2018-12-20

**Authors:** Byungjun Kang, Jisoo Shin, Hyun-Ji Park, Chanryeol Rhyou, Donyoung Kang, Shin-Jeong Lee, Young-sup Yoon, Seung-Woo Cho, Hyungsuk Lee

**Affiliations:** 1 School of Mechanical Engineering, Yonsei University, Seoul, 03722 Korea; 2 Department of Biotechnology, Yonsei University, Seoul, 03722 Korea; 3 Division of Cardiology, Department of Medicine, Emory University School of Medicine, Atlanta, GA 30322 USA; 4 Severance Biomedical Science Institute, Yonsei University College of Medicine, Seoul, 03722 Korea; 5 Center for Nanomedicine, Institute for Basic Science (IBS), Seoul, 03722 Korea

## Abstract

The fabrication of functional tissues is essential for clinical applications such as disease treatment and drug discovery. Recent studies have revealed that the mechanical environments of tissues, determined by geometric cell patterns, material composition, or mechanical properties, play critical roles in ensuring proper tissue function. Here, we propose an acoustophoretic technique using surface acoustic waves to fabricate therapeutic vascular tissue containing a three-dimensional collateral distribution of vessels. Co-aligned human umbilical vein endothelial cells and human adipose stem cells that are arranged in a biodegradable catechol-conjugated hyaluronic acid hydrogel exhibit enhanced cell-cell contacts, gene expression, and secretion of angiogenic and anti-inflammatory paracrine factors. The therapeutic effects of the fabricated vessel constructs are demonstrated in experiments using an ischemia mouse model by exhibiting the remarkable recovery of damaged tissue. Our study can be referenced to fabricate various types of artificial tissues that mimic the original functions as well as structures.

## Abstract (web summary)

Engineering 3D tissues faces the challenge of adequate vascularisation for nutrient delivery and gas exchange deep inside the construct. Here the authors use surface acoustic waves to create an aligned array of blood vessels in a hyaluronic acid hydrogel and use it to improve function in a mouse hindlimb ischemia model.

## Introduction

In vitro fabrication of functional three-dimensional (3D) tissue is technically challenging but essential for the repair or replacement of impaired tissue in the fields of tissue engineering and regenerative medicine^1^. Many groups have attempted to produce artificial tissues under in vivo conditions involving the co-culture of different types of cells and regulation of growth factors^2^. Recent biomimetic studies have demonstrated that not only the biological and biochemical environments but also the mechanical attributes, including physical and structural properties, of tissues are critical for differentiation, organogenesis, and the maturation of tissue constructs^3^.

Blood vessel organization is required for the efficient growth and function of tissues^4^. Although various methods have been proposed^5–9^, reproducing a blood vessel structure that is complex and multiscale, ranging from micrometers to centimeters, remains difficult^4^. Attempts to mimic artery-like structures using scaffolds or cell sheets^8^ have revealed limitations on the fabrication of microvessels smaller than 50 μm in diameter^7^, which have important roles in capillary exchange. In a living organism, microvessels in skeletal muscles have well-aligned cellular and extracellular structures with major paths and branches^10^. For example, the main vessels of the limb skeletal muscle develop collaterally in 3D to enable efficient and adequate perfusion to the distal leg and foot^11^. In the microvessels, endothelial cells (ECs) and other cells such as pericytes are arranged in high proximity to form blood conduits^12^. The capillary density in human skeletal muscles is in the range of 100–1000 capillaries per mm^2^^13,14^. A distance between capillaries is estimated to be ~30-100 μm, which is advantageous for diffusion^15^. Co-culture of endothelial and stromal cells promoted the formation of homogeneous microvessels by inducing the self-organization of capillaries^6,7,9^. However, this technique was limited in its ability to regulate the orientation and local distribution of vessels in the vascular tissue^7^. Three-dimensional templating^5,7^ and direct cell printing techniques^4,6^ are advantageous for producing geometry-controlled vasculatures. However, the disadvantages of these approaches include applicable biomaterials, minimum vessel size, vessel area density, and fabrication time^4,6,7^. To overcome the limitations of existing methods and fabricate vasculatures for disease treatment, it is necessary to develop a technique that comprehensively meets the following requirements: (i) 3D cellular arrangement akin to native tissue^5^, (ii) extracellular matrix (ECM) environment^3^ with clinically relevant size^16^, (iii) co-culture of multiple cell types^17^, (iv) integrated cell–cell junctions^18^, and (v) composed of biocompatible, biodegradable^19^, and tissue-adhesive^20^ biomaterials.

Recent studies have shown that pressure fields formed by standing surface acoustic waves (SSAWs) are capable of manipulating microparticles at a high resolution in a noninvasive manner^21–26^. SSAW techniques also exhibit the potential to selectively manipulate various types of microparticles^21^, regulate cell–cell distances^22^, and engineer cellular aggregates such as spheroids^25^. Such high-resolution cell engineering is essential to replicate complex and highly ordered tissues in vivo because obtaining such tissues by current methods, including bioprinting, is difficult. In this study, we introduce a tissue fabrication method by developing a cell patterning technique in a 3D hydrogel matrix using SSAW. Our method is designed to produce an implantable tissue that exhibits physiologically relevant mechanical properties, cellular density and organization. Adipose-derived stem cells and endothelial cells are co-aligned into collateral cylindroids in a biocompatible, biodegradable, and tissue-adhesive catechol-conjugated hyaluronic acid (HA-CA) hydrogel. Enhanced gene expression and growth factor secretion by the tissue fabricated by cell patterning are assessed. The therapeutic potential of 3D-patterned collateral microvessels is tested by performing in vivo implantation using a mouse model of critical limb ischemia. Our methods and results can be applied to fabricate various types of functional tissue constructs mimicking native tissue with improved regenerative efficacy.

## Results

### Fabrication of vascular tissue for ischemia therapy

**Fig. 1:**
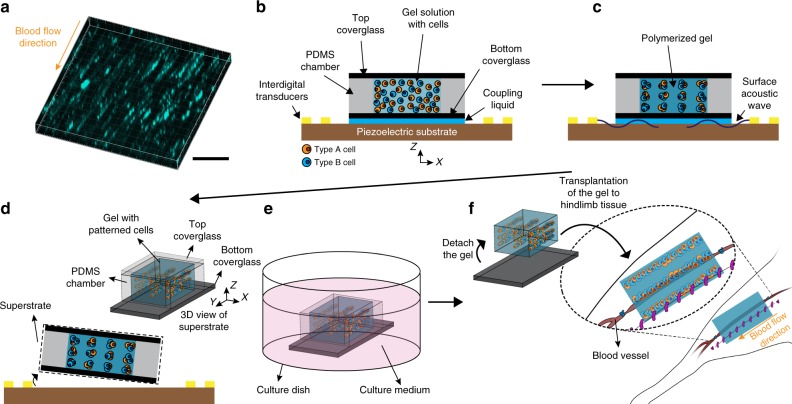
Fabrication of tissue construct with cells patterned using SSAW for ischemia therapy. **a** Fluorescent image of 70 kDa FITC-dextran in collateral vessels in mouse hindlimb tissue. Scale bar = 200 μm. **b** Injection of HA-CA hydrogel/cell mixture solution into a PDMS chamber on a piezoelectric substrate. **c** 3D cell patterning in gel solution using surface acoustic waves. **d** Disassembly of the superstrate of the hydrogel with patterned cells from the piezoelectric substrate. **e** Culturing hydrogel-cell constructs in medium. **f** Detachment of hydrogel constructs with patterned cells from the glass and transplantation of the constructs into the mouse hindlimb. The size of construct for in vivo experiments is 6, 10, and 0.53 mm in *X*, *Y*, and *Z* directions, respectively. For in vitro experiments, the size of construct is 6, 6, and 0.53 mm in *X*, *Y*, and *Z* directions, respectively

To replicate the structure of the aligned vasculatures in skeletal muscles (Fig. 1a), our acoustophoretic fabrication system was designed to arrange cells into collateral cylindrical patterns at intervals similar to the inter-capillary distance of human skeletal muscle in a 3D hydrogel matrix. To maximize the therapeutic effect of the fabricated tissue with collateral cylindroids in the mouse ischemia model, the size of the hydrogel matrix was determined to be similar to that of a mouse hindlimb muscle.

The vasculatures in the skeletal muscle primarily consist of ECs and mural cells such as pericytes or vascular smooth muscle cells (vSMCs). However, those primary mural cells requiring blood vessel biopsy are not readily accessible cell sources in clinical setting^27^. Adult stem cells exhibit angiogenic effects leading to vascular regeneration. In particular, human adipose-derived stem cell (hADSC) can be acquired in large numbers from human adipose tissue and easily expanded, which is more adequate for clinical approach. More importantly, over last few decades, ADSC has been highlighted as a highly functional therapeutic cell source for neovascularization because of its superior paracrine ability to secrete various angiogenic factors^28^. Previous studies have revealed that ADSCs have the potential to differentiate into pericyte- or vSMC-like cells^29,30^. Therefore, hADSCs were co-patterned with human vascular ECs to improve vessel formation and maturation through paracrine signaling^29,31^. Those cells were assembled to form cell–cell junctions at the pressure nodes of the standing pressure field^22^. For the hydrogel, we chose HA-CA owing to several advantages. HA itself has cellular adhesion motifs to interact with binding receptors existing in some cell types including neural stem cells and mesenchymal stem cells^32,33^. And catechol-medicated crosslinking system of HA-CA provides biocompatibility and excellent tissue adhesiveness. The biophysical and mechanical properties of HA-CA are readily adjustable for various applications, as tested previously by our group^34,35^.

The acoustophoretic fabrication system consisted of a detachable superstrate and the underlying piezoelectric substrate. The superstrate composed of a coverglass, and a polydimethylsiloxane (PDMS) chamber was placed on the substrate. The gap between the superstrate and substrate was filled with a coupling liquid. HA-CA solution mixed with cells was injected into the chamber and enclosed by the coverglass (Fig. 1b). The cells in the solution were patterned into collateral cylindroids at defined intervals in the lateral and vertical directions (*X* and *Z* directions), which replicated the architecture of vessels in hindlimb muscle (Supplementary Fig. 1), by exerting surface acoustic waves which were generated from interdigital transducers (IDTs) and propagated into the gel solution through the coupling liquid (Fig. 1c). As the HA-CA conjugate polymerized, the cells were immobilized at the patterned locations. We arranged the cells uniformly across the entire hydrogel at a clinically relevant scale (Supplementary Fig. 2). The superstrate, including the fabricated tissue, was detached from the substrate and incubated for 1 week after removing the top coverglass (Fig. 1d, e, Supplementary Fig. 3a). After removing the PDMS chamber (Supplementary Fig. 3b), the cell-patterned hydrogel was separated from the bottom coverglass (Fig. 1f, Supplementary Fig. 3c) and transferred onto the hindlimb muscle of a mouse ischemia model (Fig. 1f). We used a fine spatula to transfer the 3D tissue construct from culture substrate to animal model because of difficulty in handling due to its small dimension and low stiffness.

### Design of the acoustophoretic tissue fabrication system

**Fig. 2:**
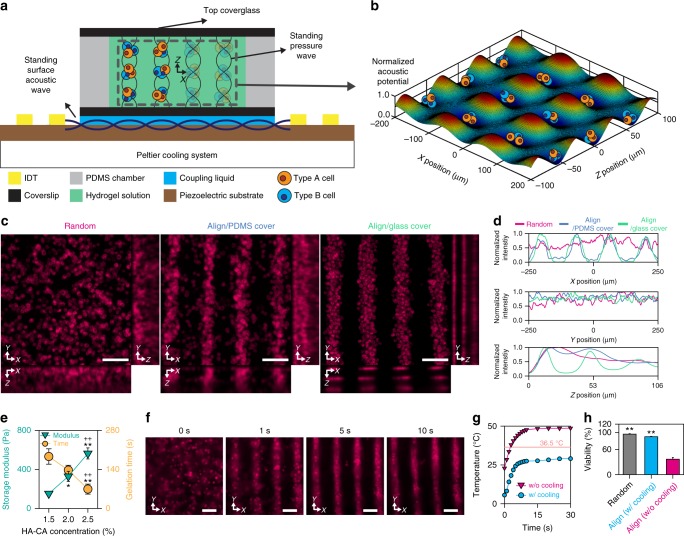
Acoustophoretic tissue fabrication system. **a** SSAW system consisting of a piezoelectric substrate, a PDMS chamber, a Peltier cooling system, and two glass coverslips designed to create a pattern of multi-type cells in a 3D hydrogel matrix. **b** 3D plot of the acoustic potential field normalized by the maximum value in the center region of the solution. **c** *XY*, *YZ*, and *XZ* projection images from confocal micrographs of DiI-stained cells in hydrogels from the Random group, Align/PDMS cover group and Align/glass cover group. Scale bars = 100 μm. **d** Normalized fluorescence intensity of cells in the *X*, *Y*, and *Z* directions in the gel. **e** Storage modulus at 1 Hz (cyan triangle, *n* = 3) and gelation time (orange circle, *n* = 4) of HA-CA hydrogel at various concentrations (technical repeats = 2, *p < 0.05 and **p < 0.01 versus 1.5% HA-CA group and ^++^*p* < 0.01 versus 2.0% HA-CA group, via one-way ANOVA followed by Tukey’s multiple comparisons test). **f** Time-lapse images of DiI-stained cells patterned by the SSAW system in 2% HA-CA hydrogel at an input voltage of 90 mVrms. Scale bars = 100 μm. **g** Change in temperature of 2% HA-CA hydrogel solution during the application of the SSAW with (blue circle) and without (magenta triangle) the cooling system. **h** Cell viability of random and aligned cells in hydrogels prepared with and without the cooling system (*n* = 3–4, technical repeats = 3, **p < 0.01 versus Align without cooling group, via one-way ANOVA followed by Tukey’s multiple comparisons test). Error bars represent one standard deviation

A primary component of the acoustophoretic tissue fabrication system is the SSAW-based cell patterning device that enables the cells to be organized into a 3D cylindroid array at high resolution. Briefly, the SSAW is created by the superposition of opposing traveling surface acoustic waves generated by exerting alternating current (AC) electrical signals to IDTs (Fig. 2a). In the coupling liquid above the piezoelectric substrate, a bulk acoustic wave (BAW) is produced perpendicular to the SSAW; this BAW has a displacement amplitude that is largest at the antinodes of the SSAW with intervals that are half of the SSAW wavelength^36^. The generated BAW is transmitted to the hydrogel solution inside the chamber via the coupling liquid and bottom coverglass and is then reflected by the chamber cover. Superposition of the transmitted BAW and its reflection by the chamber cover creates standing pressure fields in the *XZ*-plane (Fig. 2b), resulting in a 3D acoustic potential. Therefore, cells in the solution can be patterned both horizontally and vertically, with spacing determined mainly by the wavelength of SAW and that of the pressure wave in the gel, respectively^22,37^.

To implement the device, the materials for the cover and the side chamber should be properly chosen. The pattern of the acoustic potential along *Z* direction became less distinct as decreasing the reflection coefficient of the top cover (Supplementary Fig. 4a). Consequently, when PDMS (intensity reflection coefficient = 0.04) was used as the top cover, cells were rarely patterned along the *Z* direction (Fig. 2c, d). When a glass (intensity reflection coefficient = 0.56) was used, the cells were arranged in both *X* and *Z* directions more clearly (Fig. 2c, d). Additionally, the wave derived from the bottom coverglass can transmit into the top cover through the side chamber. The wave transmitted into the top cover may be reflected at the cover/air interface and propagate into the solution, disturbing the acoustic field in the solution (Supplementary Fig. 4b). The effect of the wave from the side chamber is minimized by selecting a material with a high attenuation coefficient (Supplementary Fig. 4b). Thus, PDMS was chosen as the side chamber material due to its high attenuation coefficient and ease of fabrication.

Successful regenerative medicine involving the transplantation of artificial tissues requires not only optimization of the tissue fabrication system but also the proper selection of biomaterials. Our system was applied to various ECM hydrogels with different crosslinking mechanisms (e.g., collagen, HA-CA, and methacrylate-conjugated HA (HA-MA)) (Supplementary Fig. 5a). In this study, we chose HA-CA hydrogel due to the viability and functionality of the embedded cells, its ability to maintain a cellular arrangement, biodegradability, tissue adhesiveness, and therapeutic ability to repair tissue defects as reported in the liver and hindlimb^34,35^. Cells subjected to an acoustic field experience not only an acoustic radiation force but also a drag force produced by the viscosity of the medium. As the viscosity increases during polymerization of the HA-CA hydrogel through oxidative crosslinking chemistry, SSAW is applied to the hydrogel-cell mixture with oxidant immediately after being transferred to a chamber.

The concentration of the HA-CA hydrogel was determined by taking the rheological properties of the gel and the alignment degree of patterned cells into account. To maintain the cell arrangement as patterned, a higher concentration was advantageous because the storage modulus of the solidified HA-CA hydrogel increased with the concentration (Fig. 2e, Supplementary Fig. 5b). However, as the concentration increased, it became difficult to manipulate the cells to make the patterns due to the reduced gelation time and the increased complex viscosity of the polymerizing hydrogel solution (Fig. 2e, Supplementary Fig. 5c). Thus, we chose 2% HA-CA as the optimal concentration, with a gelation time of 154.8 s and an initial complex viscosity of 0.13 Pa·s. We also found that the mechanical response of 2% HA-CA to applied forces was similar to that of mouse hindlimb muscle tissue in indentation tests (Supplementary Fig. 5d).

In fact, necrotic cell death often occurs in the center of large and dense cellular structure due to diffusional limitation of nutrients and oxygen supply. However, the HA-CA hydrogel is highly permeable to molecules in aqueous solution due to its swelling behavior and microporous structure^34^. The high permeability of HA-CA hydrogel was beneficial to efficient mass transfer of fabricated 3D construct for supply of nutrients/oxygen and removal of metabolic wastes, resulting in maintaining cell viability and minimal necrosis in our experiments.

Controlling the temperature is important when fabricating tissues using the acoustophoretic system because attenuation of the pressure wave in the hydrogel increases the temperature, which can reduce cell viability^38^. We noted that the average temperature of deionized water in the chamber increased by an input voltage applied to the system (Supplementary Fig. 6a). When the input voltage was higher than 60 mV_rms_, the temperature was higher than 36.5 °C, impacting cell survival. To prevent cell death, our system was equipped with a Peltier-based cooling device (Supplementary Fig. 6b). Under our experimental condition (90 mV_rms_), the duration of the SSAW application for cell patterning was less than 10 s which was one order shorter than the gelation time of the 2% HA-CA hydrogel (Fig. 2e, f). The temperature of the hydrogel was kept below 30 °C (Fig. 2g, Supplementary Fig. 6c), and the cell viability was around 90% (Fig. 2h, Supplementary Fig. 6d). Thus, we were able to fabricate a tissue construct without significant damage to cells using our established system.

### A vascular construct replicating vessels in skeletal muscle

**Fig. 3:**
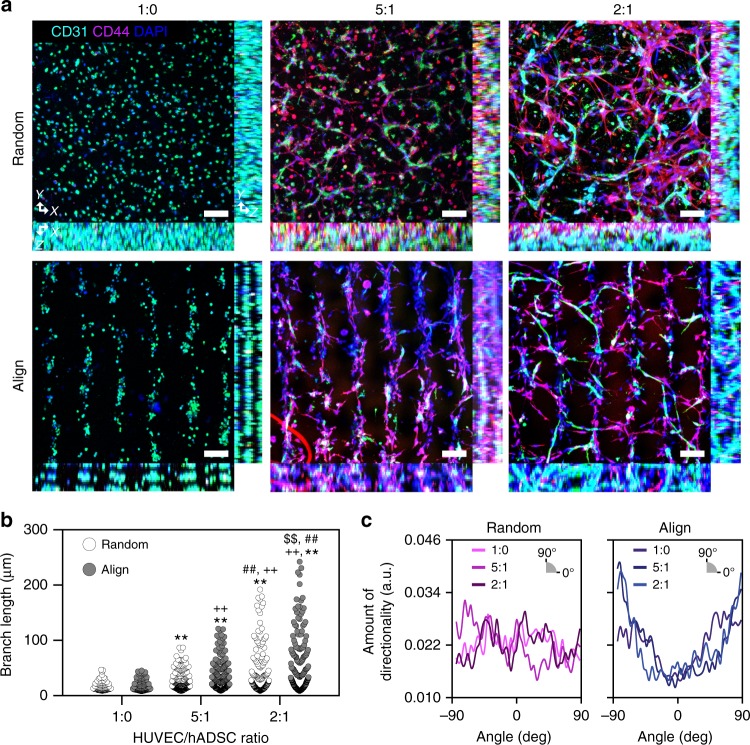
SSAW-induced 3D cell patterning for the recapitulation of microvessels in hindlimb muscle. **a** Dual immunofluorescence staining of CD31 for HUVECs and CD44 for hADSCs at various HUVEC/hADSC ratios (1:0, 5:1, 2:1—total cell density: 1 × 10^7^ cells ml^−1^) 1 week after cultivation (technical repeats = 3). Scale bars = 100 μm. Fluorescent image-based quantification of **b** HUVEC elongation (*n* = 92–247) and **c** elongated direction using CD31-stained images (technical repeats = 2, **p < 0.01 versus 1:0 Random group, ^++^*p* < 0.01 versus 1:0 Align group, ^##^*p* < 0.01 versus 5:1 Random group, and ^$$^*p* < 0.01 versus 2:1 Random group, via one-way ANOVA followed by Tukey’s multiple comparisons test)

Here, we recapitulated 3D collateral vessel structure in the hindlimb muscle by performing 3D patterning of human stem cells and ECs in lines within an HA-based hydrogel. Without the SSAW application, the cells were randomly distributed within the 3D hydrogel, showing an alignment index close to the minimum value (Supplementary Fig. 1). In contrast, when SSAW was applied, cells within the HA-CA hydrogel were patterned into lines organizing a collateral vessel-like structure. To obtain a continuous line pattern of cells, we determined the optimal cell density in the 3D hydrogel based on images of a ‘Random’ group and an ‘Align’ group. As the cell density increased, the difference between the ‘Random’ group and the ‘Align’ group became more significant (Supplementary Fig. 7). When the cell density was higher than 1.0 × 10^7^ cells ml^−^^1^, cells in the ‘Align’ group formed cylindroids throughout the entire hydrogel area. Given this result, the cell density was optimized as 1.0 × 10^7^ cells ml^−1^ for in vitro analysis. During angiogenesis, ECs build a vascular network and recruit mural cells to form mature and stable vessels^39^. To fabricate native microvessels with structural and paracrine supports for ECs in the angiogenic process, human umbilical vein endothelial cells (HUVECs) were co-cultured with hADSCs at various ratios (HUVEC:hADSC = 1:0, 5:1, or 2:1)^29,31^ (Fig. 3a). We maintained the total cell density same, and varied a ratio of HUVEC to ADSC density in experiments. Without hADSCs, the HUVECs maintained a circular morphology and could not extend or sprout at all within the hydrogel until one week after culturing (1:0 group). When the ratio of hADSCs was increased, the HUVECs exhibited a more stretched morphology and sprouted out (5:1 group versus 2:1 group) (Fig. 3a). HUVECs that were co-cultured with hADSCs in the SSAW-induced groups were elongated in a guided direction following a line pattern (Fig. 3b, c).

### Improved vascular function and maturation of patterned cells

**Fig. 4:**
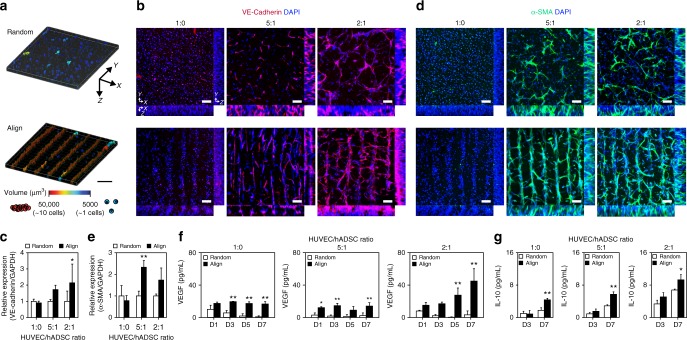
Effects of enhanced cell–cell contact in the fabricated cell-hydrogel construct on vessel maturation. **a** Reconstructed 3D images of contacting cells within the hydrogel. Scale bar = 150 μm. **b** Representative fluorescent images showing the expression of the EC-specific cell junction marker VE-cadherin at various HUVEC/hADSC ratios (1:0, 5:1, 2:1—total cell density: 1 × 10^7^ cells ml^−1^) (technical repeats = 3). Scale bars = 100 μm. **c** qPCR analysis to quantify the expression of VE-cadherin in aligned/randomly distributed cells within the hydrogel (*n* = 3–6, technical repeats = 2, *p < 0.05 versus each Random group, via two-way ANOVA followed by Sidak’s multiple comparisons test). **d** Immunofluorescence staining of cell-hydrogel constructs with the mural cell marker α-SMA (technical repeats = 3). Scale bars = 100 μm. **e** qPCR analysis to quantify the expression of α-SMA in aligned/randomly distributed cells within the hydrogel (*n* = 3–6, technical repeats = 2, **p < 0.01 versus each random group, via two-way ANOVA followed by Sidak’s multiple comparisons test). **f** ELISA analysis of VEGF secretion by aligned/randomly distributed cells in hydrogels at days 1, 3, 5, and 7 (*n* = 3, technical repeats = 2, *p < 0.05 and **p < 0.01 versus each Random group, via two-way ANOVA followed by Sidak’s multiple comparisons test). **g** ELISA analysis of IL-10 secretion by aligned/randomly distributed cells in hydrogels at days 3 and 7 (*n* = 3–4, technical repeats = 2, *p < 0.05 and **p < 0.01 versus each Random group, via two-way ANOVA followed by Sidak’s multiple comparisons test). Error bars represent one standard deviation

With respect to vessel maturation, contact and communication between EC-EC and EC-mural cells are critically important for tight junction formation, vascular permeability, and growth factor-dependent EC survival and stabilization^18^. Therefore, we expected the SSAW-mediated alignment of the 3D vasculature composed of HUVECs and hADSCs to promote the functional maturation of vessels by increasing the local cell density and enhancing cell–cell contacts (Fig. 4a, Supplementary Fig. 7). Enhanced interactions between HUVECs and HUVECs-hADSCs induced a significant increase in the expression of vascular endothelial (VE)-cadherin, which is an EC-specific adhesion molecule that has a critical role in vascular maturation^40^ (Fig. 4b, c). Formation of tight junctions between HUVECs was also observed to be upregulated in 3D-aligned HUVECs with hADSCs as characterized and compared from the immunostained images of Zonula occludens-1 (ZO-1), a marker of tight junctions, indicating the important role of increased cell–cell contacts in formation of functional endothelium with tight junction (Supplementary Fig. 8a, b). Furthermore, the formation of vascular lumen-like structure was observed within 3D-aligned construct at HUVEC/hADSC 2:1 ratio, as shown in ortho-view of magnified images immunostained by ZO-1 (Supplementary Fig. 8c). In addition to upregulated expression of VE-cadherin, we confirmed that increased contact between HUVECs and HUVECs-hADSCs in SSAW-applied groups upregulated gene expression of Tie2 and von Willebrand Factor (vWF), which are highly related to vascular maturation^41^, especially in co-cultured groups at 2:1 ratio of HUVEC/hADSC (Supplementary Fig. 9a). Furthermore, increasing direct cell–cell contact between HUVECs and hADSCs via SSAW-induced 3D alignment upregulated the expression of a mural cell marker—smooth muscle alpha-actin (α-SMA)—in hADSCs (Fig. 4d, e). Thus, α-SMA-expressing hADSCs in 3D-patterned vascular structures may have a mural cell-like role in microvessel maturation and stabilization^42^.

Furthermore, platelet-derived growth factor (PDGF) secretion of HUVECs, which has a critical role in the maturation of vessel through the recruitment of pericytes^43^, was promoted in SSAW-applied co-cultured groups, due to the enhanced cell–cell interactions (Supplementary Fig. 9b). hADSCs have been regarded as an attractive therapeutic cell source for ischemic diseases due to their paracrine ability^44^. hADSCs have shown immune-modulating effects that attenuate ischemia-induced inflammation by secreting anti-inflammatory factors, in addition to exerting angiogenic effects^45^. Therefore, the levels of angiogenic factors (vascular endothelial growth factor; VEGF) and anti-inflammatory cytokines (interleukin-10; IL-10) secreted from both aligned and randomly distributed cells within HA-CA hydrogels at various HUVEC/hADSC ratios (1:0, 5:1, and 2:1) were compared on days 1, 3, 5, and 7 after encapsulation (Fig. 4f, g). Increased cell–cell contacts in the aligned groups through acoustophoretic patterning resulted in a significantly higher secretion of VEGF and IL-10 compared to that in the random groups, contributing to enhanced angiogenesis and decreased apoptosis at ischemic defect sites. By increasing the number of hADSCs, more extensive secretion of VEGF and IL-10 was detected. Taken together, HUVEC/hADSCs co-cultured at a 2:1 ratio were expected to show the greatest therapeutic potential for angiogenesis and tissue healing when they were aligned and condensed in 3D collateral vessel-like structures within the hydrogel.

To demonstrate the applicability of our methodology further, we fabricated a 3D vascular construct using human induced pluripotent stem cell (hiPSC)-derived EC (hiPSC-EC), instead of HUVEC. hiPSC-ECs were well-aligned with hADSCs (Supplementary Fig. 10a) and they exhibited more branches along the cell alignment compared to random group (Supplementary Fig. 10b, c). This result shows a potential that our technique can be applied to a clinical therapy which requires patient-specific autologous cells^46^.

### Integration of vascular constructs into host vasculature

**Fig. 5:**
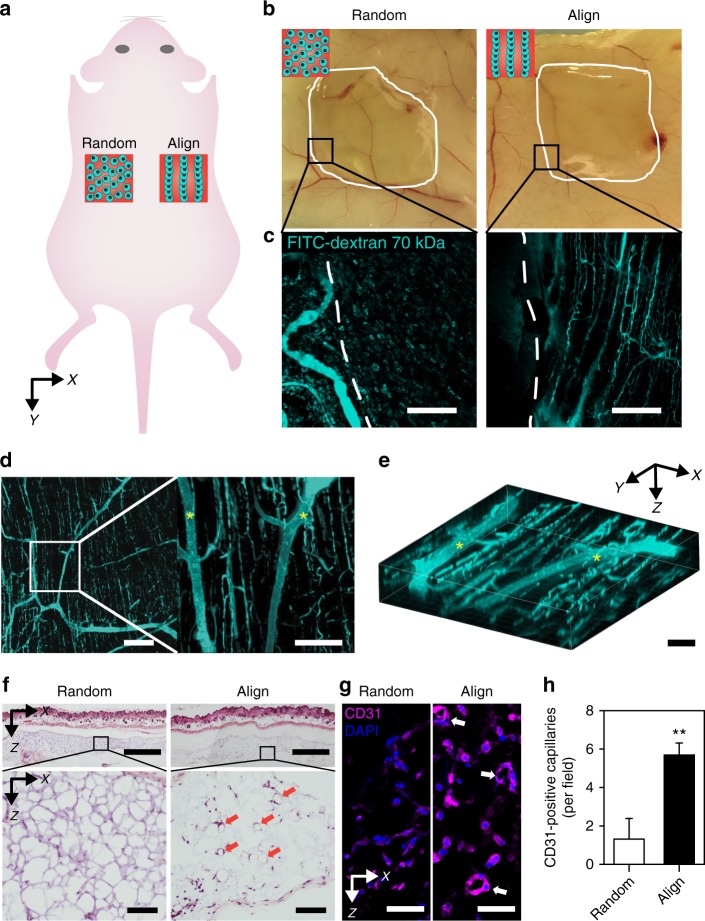
Integration of patterned microvessels with the host vasculature in transplantation. **a** Schematic illustration showing the transplantation of randomly distributed and aligned cell-hydrogel constructs into the subcutaneous space of the mouse back. **b** Gross view of harvested back-skin tissues with cell-hydrogel constructs 1 week after transplantation. **c** Fluorescent images of 70 kDa FITC-dextran-perfused vessels in one part of the image (**b**). Scale bars = 200 μm. Representative **d** 2D images and **e** 3D image of FITC-dextran-labeled vessels in transplanted 3D-aligned cell-hydrogel constructs (HUVEC/hADSC ratio at 2:1—total cell density: 1.2 × 10^7^ cells ml^−^^1^) 1 week after transplantation. The asterisks indicate infiltrated host vessels. Scale bars = 200 μm for the left image of **d**, 100 μm for the right image of **d**, and 50 μm for **e**. **f** H&E-stained images of cross-sectioned 3D cell-hydrogel constructs with adjacent skin tissue 1 week after transplantation. Red arrows indicate vessel-like structures. Scale bars = 500 μm (upper image) and 200 μm (lower image). **g** Immunofluorescence staining of CD31 in a 3D cell-hydrogel construct 1 week after transplantation. White arrows indicate CD31-positive capillaries with lumen structure. Scale bars = 50 μm. **h** Quantification of CD31-positive capillary density in a 3D cell-hydrogel construct 1 week after transplantation (*n* = 3, technical repeats = 2, **p < 0.01 versus Random group, via unpaired *t*-test). Error bars represent one standard deviation

To examine the integration of the engineered 3D vascular construct with aligned HUVECs/hADSCs at a 2:1 ratio into the host vasculature, the constructs were transplanted subcutaneously into the dorsal regions of mice (Fig. 5a). A subcutaneous pocket on the back of the mouse was chosen as the transplantation site because this region is one of the best in vivo sites to examine vascular connections and integration into host vasculature^47^, and transplanted hydrogel constructs can remain intact. Fluorescein isothiocyanate (FITC)-conjugated dextran (FITC-dextran, 70 kDa) was perfused via tail vein injection at day 7 post-transplantation, and then the transplanted 3D vascular constructs were imaged with the surrounding skin tissue (Fig. 5b–e). Surprisingly, the extensive FITC-dextran perfusion of capillaries was observed only with the 3D constructs showing parallel lines of cellular alignment within the HA-CA hydrogel (Fig. 5c–e). The host vessels infiltrated into the transplants and further branched into engineered capillaries, which had longitudinal alignment (Fig. 5d, e). These results indicated that 3D-patterned alignment in the hydrogel construct promoted the formation of perfusing microvessels under guided direction by facilitating the direct integration of transplanted vessel structures into the native host vasculature. It occurred because aligned ADSCs attracted and guided native blood vessels toward the alignment direction of the transplanted tissue via paracrine secretion of various angiogenic factors^48^. This phenomenon indicates that patterned cells not only mimic the vessel-specific structures, but also have an important role in integrating with native vessels by spatially regulating angiogenic signaling.

Histological analyses of the 3D vascular construct with adjacent skin tissue revealed aligned microvessel-like structures within the hydrogel network. In contrast to the randomly distributed cell-hydrogel construct, the aligned group showed a larger number of vessel-like structures with lumens in the hydrogel network (Fig. 5f). Immunohistochemical staining for CD31 revealed that the observed vessel-like structures in hematoxylin & eosin (H&E)-stained images were primarily CD31-positive capillaries (Fig. 5g). As expected, the larger number of CD31-positive capillaries was detected in aligned group (5.7 ± 0.8 capillaries per field, mean ± one standard deviation) than in random group (1.3 ± 1.3 capillaries per field) (Fig. 5h). At 3 weeks after subcutaneous transplantation, the HA-CA hydrogels with aligned structures degraded more rapidly, whereas the hydrogels with random structures remained fully intact (Supplementary Fig. 11a). These results may indicate that ECM remodeling in the transplanted hydrogel construct is accelerated by a 3D vascular arrangement that promotes the formation of perfused vessels and host vascular integration. Importantly, at 3 weeks after transplantation, the development of numerous well-aligned collateral microvessels that were connected to host vessels was observed at sites of aligned hydrogel degradation in skin tissue, whereas both vessel formation and host vessel integration were rarely observed at the sites of random hydrogel transplants, and host vessel integration was also not observed (Supplementary Fig. 11b). Taken together, our results clearly showed that 3D-aligned vascular constructs fabricated by SSAW produced geometrically controlled and functional neovascularization that integrated with the host vasculature.

### Therapeutic angiogenesis by vascular constructs

**Fig. 6:**
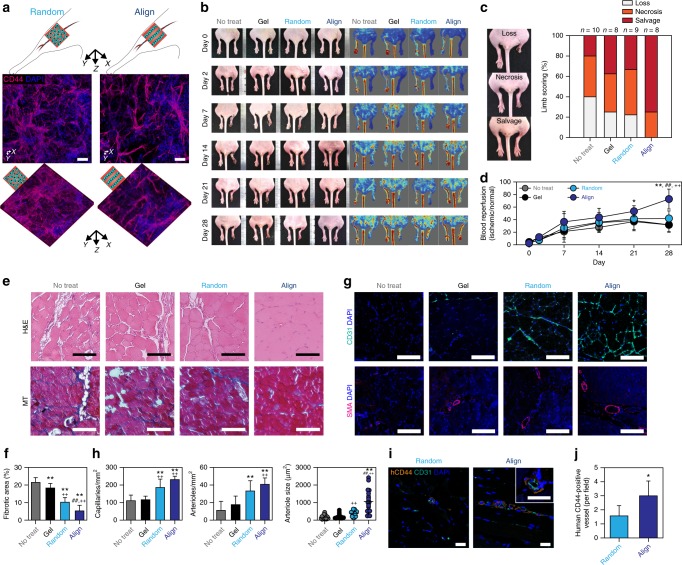
Enhanced therapeutic angiogenesis via transplantation of the fabricated cell-hydrogel construct. **a** Immunofluorescence staining of CD44 for determining the maintenance of cell alignment in retrieved cell-hydrogel constructs (HUVEC/hADSC ratio at 2:1—total cell density: 1.2 × 10^7^ cells ml^−1^) 3 days after transplantation into ischemic hindlimb of mouse. Scale bars = 100 μm. **b** Representative serial photographs (left) and blood perfusion images (right) of ischemic hindlimb at days 0, 2, 7, 14, 21, and 28 after cell transplantation. **c** Scoring for the physiological status of ischemic limbs on day 28. **d** Quantification of the relative blood perfusion rate of ischemic limb to normal limb in each group (*n* = 7–8, technical repeats = 2, *p < 0.05 and **p *<* 0.01 versus No-treatment group, ^++^*p* < 0.01 versus Gel group, and ^##^*p* < 0.01 versus Random group, via two-way ANOVA followed by Sidak’s multiple comparisons test). **e** H&E (upper) and Masson’s trichrome (lower) staining of the cross-sectioned ischemic muscles 28 days after cell transplantation. Scale bars = 100 μm. **f** Quantification of fibrotic area in the ischemic site (*n* = 10, technical repeats = 2, **p < 0.01 versus No-treatment group, ^++^*p* < 0.01 versus Gel group, ^##^*p* < 0.01 versus Random group, via one-way ANOVA followed by Tukey’s multiple comparisons test). **g** Immunofluorescence staining of CD31 (capillaries; upper) and α-SMA (arterioles; lower) in the cross-sectioned ischemic muscles 28 days after cell transplantation. Scale bars = 100 μm. **h** Quantification of CD31-positive capillary density (*n* = 7, left), α-SMA-positive arteriole density (*n* = 7, middle) and size (*n* = 20, right) in ischemic limb tissue (technical repeats = 2, **p < 0.01 versus No-treatment group, ^++^*p* < 0.01 versus Gel group, and ##*p* < 0.01 versus Random group, via one-way ANOVA followed by Tukey’s multiple comparisons test). **i** Dual immunofluorescence staining with human-specific anti-CD44 (for transplanted hADSCs) and mouse/human-co-reactive anti-CD31 (capillaries) in the cross-sectioned ischemic regions 28 days after cell transplantation. Scale bars = 20 μm. **j** Quantification of human CD44-positive vessels per field in ischemic limb tissue (*n* = 7–10, technical repeats = 2, *p < 0.05 versus Random group, via unpaired *t*-test). Error bars represent one standard deviation

Before investigating the therapeutic efficacy of fabricated vascular constructs, we confirmed whether the 3D alignment of cells in HA-CA hydrogel (2:1 ratio of HUVEC/hADSC) was maintained even after transplantation into ischemic muscle in a mouse model of hindlimb ischemia. Aligned and randomly distributed cell-hydrogel constructs were transplanted into ischemic hindlimbs of mice and retrieved at day 3 post-transplantation. Immunohistochemical staining of the retrieved hydrogels for the detection of an hADSC marker (CD44) indicated that the 3D-aligned structure in the transplanted HA-CA hydrogel construct was well-preserved in vivo (Fig. 6a).

Finally, we assessed the therapeutic efficacy of 3D-aligned cell-hydrogel constructs formed by SSAW induction by transplanting the constructs, which were pre-matured in vitro for 1 week, into a mouse model of hindlimb ischemia^49^. Twenty-eight days after transplantation, the aligned group showed significantly improved ischemic limb salvage (75%) with reduced loss of feet and limbs and reduced necrosis (Fig. 6b, c). Post-ischemic blood flow recovery was sequentially analyzed by using a Doppler imaging technique over 4 weeks. At four weeks after transplantation, 72.8% of blood flow was restored in the aligned group, whereas only 31.6%, 31.8%, and 42.0% recovery of blood perfusion was observed in the no-treatment, hydrogel-alone, and random groups, respectively (Fig. 6b, d). The native microvessel-mimicking vascular structure, which contains collaterally aligned ECs and supporting stromal cells and exhibits matured vascularization via enhanced direct cell–cell contact, may contribute to improved blood perfusion and salvage of the ischemic limb. In histological analyses performed 4 weeks after transplantation (Fig. 6e, g), H&E and Masson’s trichrome staining indicated that muscle fiber degeneration and fibrosis in ischemic muscles were significantly prevented in the aligned group compared with other control groups (no-treatment, hydrogel-alone, and random) (Fig. 6e, f) due to the anti-inflammatory and angiogenic effects confirmed in in vitro experiments. Neovascularization was evaluated by immunohistochemical staining of CD31-positive capillaries and α-SMA-positive arterioles in ischemic muscles (Fig. 6g). The aligned group exhibited a significant increase in the densities of newly formed capillaries and arterioles, and the average size of arterioles was higher than that in the other groups (Fig. 6h). More importantly, immunohistochemical staining of human-specific CD44 for the detection of hADSCs and CD31 for the detection of capillaries indicated that there were larger numbers of blood vessels originating from human cells in the aligned group than in the random group (Fig. 6i, j). These results clearly indicated that the transplantation of 3D-aligned vascular cell-hydrogel constructs generated by SSAW contributed to highly effective functional blood vessel formation, leading to improved therapeutic angiogenesis for the treatment of critical peripheral ischemic diseases.

## Discussion

Our acoustophoretic technique overcomes the limitations of conventional microvessel fabrication techniques in terms of fabrication time, material selection, and cell manipulation resolution^4,6,7^. The duration required for tissue fabrication in our method is similar to the gelation time of the hydrogel because the cell patterning occurs in less than 10 s. In addition, our patterning technique is advantageous for fabricating various types of tissues due to its versatility, as demonstrated in experiments using hydrogels with various crosslinking systems (Supplementary Fig. 5a). The resolution of our technique can be estimated by measuring distances between cell patterns and width of aligned cell patterns. The average of *X*-directional interval was measured to be 64, 124, and 132 μm depending on the IDT pattern. These values are very similar to the half wavelength of applied SAW, which were 62.5, 125, and 140 μm, respectively. The *Z*-directional interval was also varying in the range of 17–37 μm. However, the ratio of *Z*-directional interval to X-directional one was not significantly varied independent of SAW wavelength (Supplementary Fig. 12a, b). Considering the wavelength of conventional SAW devices^36^, the intervals between cell patterns can be modulated in the rage of few microns to hundreds microns. A width of aligned cell pattern was characterized as a function of cell density. It increased from 25 to 51 μm when the cell density increased from 1 × 10^6^ to 2 × 10^7^ cells ml^−1^, respectively (Supplementary Fig. 12c). Physical contact between cells patterned by our method is ensured due to the attractive secondary Bjerknes forces generated by the scattering of acoustic field^50^. As a result, it can help to enhance the cell–cell communication. The control of cell aggregation was not readily obtained in 3D printing methods^51^.

In addition to the simple line-shaped cell pattern in this study, it is possible to make more complicated patterns by using IDTs of various shapes. For example, when SSAW is generated by bi-directionally arranged IDTs, cells can exhibit alignments in the lateral, horizontal, and vertical directions (*X*, *Y*, and *Z* directions) (Supplementary Fig. 12d). When SSAW is combined with other techniques such as the frequency modulation using chirped IDTs^52^ or the phase modulation^21^, it can be possible to arrange different types of microparticles or cells at distinct locations (Supplementary Fig. 13). In conclusion, our method can be applied to fabricate various tissues with more complicated organization, such as neuro-muscular and epithelial-endothelial junctions.

Two approaches for therapeutic transplantation for ischemia have been suggested: stem cells^6,7,9^ and pre-aligned microvessel constructs^5^. In the former, angiogenesis and tissue recovery are promoted by paracrine effects and vascular differentiation. However, formation of the vessel network requires weeks, and the structures of the vessels are random and immature^6,7,9^. The microvessels formed by stem cell therapy are often unstable and do not persist in the long term^53,54^. The aligned microvessels constructed by using a 3D template or direct cell printing^55^ help form functional vessels in murine ischemia, leading to a rapid recovery^5^. Mirabella et al.^5^ demonstrated the successful therapeutic application of 3D-printed vascular constructs with aligned ECs for treatment of hindlimb ischemia and myocardial infarction. However, the paracrine effects mediated by the EC constructs may not be sufficient for angiogenesis and vascular maturation. Also, the vessel area density, defined as the number of vessels in a unit area of cross section, was slightly smaller than that observed in native tissue in vivo^56^. The resolution that determines the spatial distribution of vessels may also need further improvement.

Our method is complementary, and it is able to arrange stem cells and ECs in parallel lines simultaneously. Patterning cells in both the lateral and vertical directions increased the area density of microvessels and further enhanced the paracrine effect. This effect, encompassing VEGF and IL-10 secretion induced by HUVEC/hADSC co-culture, was increased by SSAW-induced cell aggregation, resulting in the significant enhancement of angiogenesis and tissue recovery. In addition, improvement of vascular stability and maturation by patterned HUVEC/hADSC co-culture induced the integration of the 3D microvessel networks into the host vasculature and produced perfusable microvessels. Due to the effective transport of blood to the end of the leg through the large arterioles and microvessels produced at the centimeter scale^5,57^, the transplanted tissue constructs exhibited a significant increase in the rate of tissue recovery in the ischemic model. Nonetheless, our SSAW-based 3D patterning will be further improved by future studies investigating (i) the biophysical effects of cylindrical cell arrangement and inter-cylindroid spacing on cell maturation and vascular generation and (ii) the mechanism of vascular integration between implanted vessels and host vessels in vivo. We expect that our therapeutic 3D vascular tissue will be effective not only for the treatment of human vascular diseases but also other diseases such as organ malperfusion^5^.

Each organ in the body has its own vasculature with distinctive functions^15,58^. Common and unique functions of the vasculature in each organ are accomplished by organ-specifically differentiated ECs^15,58^. Therefore, the tissue-specific structure of ECs should be taken into account in producing the organotypic vasculature using the acoustophoreic cell patterning system. Also, the lymphatic vascular systems are closely interconnected with blood vascular system^59^. It is essential for maintaining tissue fluid homeostasis, fat absorption and immunological surveillance^59^. In order to fabricate a more physiologically relevant vascular tissue, it is probably required to engineer both blood and lymphatic vessels in one tissue construct.

In conclusion, we developed a SSAW-based 3D cell-patterning technique to fabricate a therapeutic tissue consisting of aligned microvessels organized at a high resolution. The fabricated tissue exhibited the upregulated expression of cell–cell junctions and mural cell markers and the increased secretion of angiogenic factors and anti-inflammatory cytokines. The transplanted 3D vascular construct formed perfusable and aligned vessels in mouse models and showed therapeutic angiogenesis in a hindlimb ischemia mouse model. Our study describes an innovative method - a 3D acoustophoretic cell patterning technique utilizing a biocompatible hydrogel - to engineer functional tissues exhibiting in vivo-like structural features and regenerative efficacy for therapeutic applications and drug testing.

## Methods

### Preparation of the SSAW device

The SSAW device was composed of a piezoelectric substrate and IDTs. Two sets of IDTs consisting of a 2-μm-thick aluminum layer were deposited and patterned on a 500-μm-thick 128° Y-cut lithium niobate wafer by a conventional photolithography technique^22^. The width of the IDTs and the interval between each IDT finger were 70 μm. Each IDT set in the SSAW device was connected to an arbitrary waveform generator (33622A, Keysight Technologies, Santa Rosa, CA, USA) and a radio frequency (RF) amplifier (LZY-22+, Mini-Circuits, Brooklyn, NY, USA). The wavelength of the surface acoustic wave was 280 μm. The frequency of the SAW was 13.928 MHz.

### Superstrate preparation

Our system was designed to handle a fabricated tissue construct with ease for transplantation by allowing detachment of a superstrate containing the tissue from the substrate^26^. The detachable superstrates consisted of a coverglass (10.5 × 22 mm) and a PDMS chamber. To fabricate the detachable superstrates, PDMS solution (Sylgard 184, Dow Corning, Midland, MI, USA) with a 10:1 (mass ratio of base to curing agent) mixing ratio was first poured onto an acryl rectangular block (100 × 100 × 10 mm) with 0.53-mm-thick spacers and degassed in a vacuum pump for 1 h. Another acryl block was then placed on the solution and baked at 60 °C for 24 h. A chamber was prepared by cutting the PDMS block using a customized cutter. After cleaning and wetting the chamber using alcohol, the chamber was placed in the center of the coverglass (10.5 × 22 mm). By drying the PDMS/glass superstrate at 60 °C for 30 min, the PDMS chamber was reversibly bonded to the bottom glass by Van der Waals interactions. This allowed us to disassemble the superstrate containing the fabricated tissue for transplantation. The superstrate was sterilized under UV illumination for more than 1 h prior to the experiment. The size of the PDMS chamber was 6 × 6 × 0.53 mm for in vitro experiments and 6 × 10 × 0.53 mm for in vivo experiments. The chamber in the superstrate was covered by another coverslip.

### Preparation of a Peltier-based cooling system

The SSAW device was bonded to a flat aluminum chip using conductive epoxy to minimize the effect of bulk acoustic wave inside the piezoelectric substrate^60^. The aluminum chip was thermally coupled with a Peltier cooling system using a silicone-based thermal pad (Arctic, Braunschweig, Niedersachsen, Germany). The Peltier cooling system was composed of aluminum plate, Peltier element, heat sink, fan, temperature controller, and temperature sensor. A temperature sensor was attached to the aluminum plate, and the temperature was controlled by the proportional-integral-derivate (PID) method. The temperature change in the solution was measured by a thermocouple probe and a thermometer.

### Computational study of acoustic fields in the SSAW device

To simulate acoustic fields in our cell patterning device, a frequency domain 2D model was developed using COMSOL Multiphysics V5.3a (COMSOL, Stockholm, Sweden). Dimensions of the model were determined with considering those of the experimental device. A perfectly matched layer (PML) domain was set for the underside of the piezoelectric substrate^24^. The piezoelectric substrate domain was modeled by using the ‘Solid Mechanics’, ‘Electrostatics’, and ‘Piezoelectric Devices’ modules. An AC signal was applied to the IDTs on a piezoelectric substrate^24^. The coupling liquid domain was modeled using the ‘Pressure Acoustics’ module. The solution domain was modeled using the ‘Thermoacoustics’ module. The bottom and top coverglass domains were modeled using the ‘Solid Mechanics’ module. The chamber domain was modeled using the ‘Pressure Acoustics’ module for the PDMS chamber because the shear wave is negligible compared to the longitudinal wave in PDMS^24^. The interface between domains for pressure acoustics and those for solid mechanics was set as the ‘Acoustic-solid interaction’ boundary. To analyze the effect of reflection at the water/cover interface, the top boundary of the solution domain was set as the ‘Impedance’ boundary where the acoustic impedance was calculated from the speed of sound and density of the material. Material properties of water, glass, and lithium niobate were obtained from the COMSOL Material Library. Other parameters utilized in the computational study are listed in Supplementary Table 1 and 2.

Acoustic potential *U* on a single spherical particle, corresponding to a cell in this work, were calculated with Eq. (1):^22^

1 $${{U = V}}_{\mathrm{0}}\left[ {{{f}}_{\mathrm{1}}\left( {{{\beta }}_{\mathrm{f}}{\mathrm{/4}}} \right){{{\mathrm{Re}}}}\left( {{{p}} \cdot {{p^\ast }}} \right){{ - f}}_{\mathrm{2}}\left( {{{3\rho }}_{\mathrm{f}}{\mathrm{/8}}} \right){{{\mathrm{Re}}}}\left( {{{\nu }} \cdot {{\nu ^\ast }}} \right)} \right]$$

where *f*_1_ = 1−(*β*_p_/*β*_f_), and *f*_2_ = 2(*ρ*_p_ *– ρ*_f_)/(2*ρ*_p_ *+* *ρ*_f_). *V*_0_ is the volume of the particle. *ρ*_p_*, ρ*_f_*, β*_p_, and *β*_f_ are the density and compressibility of the particle and the fluid, respectively. *p* and *ν* are the acoustic pressure field and the acoustic velocity field inside the solution calculated from the frequency domain analysis, respectively. Re indicates the real part of the complex number. The asterisk indicates complex conjugation.

### Preparation of the HA-CA hydrogel

HA-CA conjugate was synthesized by modifying HA (MW 200 kDa, Lifecore Biomedical, Chaska, MN, USA) with a catechol group using dopamine hydrochloride (Sigma, St. Louis, MO, USA)^34,35^. In brief, HA was dissolved in distilled water at a concentration of 1% (w/v). Equal molar quantities of 1-ethyl-3-(3-dimethylaminopropyl) carbodiimide (EDC) (TCI Co., Tokyo, Japan) and N-hydroxysulfosuccinimide (NHS) (Sigma) (relative to the HA backbone unit) were then added into the solution and stirred for 30 min. Dopamine hydrochloride was added to the solution at an equal molar ratio to HA, and the pH of the solution was adjusted to 5.0 using 1 M hydrochloride. The reaction was continued overnight by stirring at room temperature. To eliminate unreacted dopamine hydrochloride, the solution was dialyzed against 1× phosphate buffered saline (PBS) (3 M Korea, Seoul, Korea) at a pH of 5.0 four times each for 6 h and against distilled water once for 4 h. The resultant solution was then frozen and lyophilized. For gelation, the HA-CA pre-gel solution (dissolved in PBS) was mixed with a sodium periodate solution (NaIO_4_, Sigma) at an equal molar ratio to catechol to oxidize the catechol groups of HA-CA to reactive o-quinones, which forms covalent crosslinks^34,35^. The pH condition of HA-CA hydrogel was adjusted to 7.2–7.4 using NaOH-containing NaIO_4_ solution for cell culture.

### Rheological analysis of the HA-CA hydrogel

The rheological properties of the HA-CA hydrogel with various gel concentrations (1.5, 2, and 2.5%) (w/v) were determined using a rheometer (MCR102, Anton Paar, Graz, Austria). The elastic moduli of the HA-CA hydrogels were measured in frequency sweep mode over 0.1–100 Hz at a constant shear strain (2%). The gelation kinetics (complex viscosity and gelation time) of the HA-CA hydrogels were investigated in time sweep mode at a constant frequency of 1 Hz and shear strain of 2%. The gelation time of the hydrogels was determined as the crossover point of the storage modulus (*G*′) and loss modulus (*G*″).

### Indentation test for mouse tissue and hydrogel

Mouse hindlimb muscle tissue and hydrogel samples were dissected by using a biopsy punch with a diameter of 4 mm and sliced to a thickness of 2 mm. A custom-made indentation instrument^61^ was utilized to measure the mechanical properties of the samples. The indentation tip was a 1-mm-diameter ball of stainless steel, and the indentation speed was 25 μm s^-1^. Indentation forces were recorded at every 2.6 μm of indentation depth. The indentation depth was larger than 130 μm. The contact points were defined by using the ‘goodness of fit’ method^62^. Briefly, 100 points of data from an arbitrary data point were fitted to the Hertzian contact model^63^ described in equation (2), and the contact point was defined as the data point when the mean-squared error of the fitting is minimized.

2 $$F = \left( {4/3} \right) \cdot E^ \ast R^{0.5}d^{1.5}$$

where *F*, *R*, and *d* are the measured force, the radius of the indentation tip, and the indentation depth, respectively. E* is defined as Eq. (3):

3 $$1/E^ \ast = \left( {1{\mathrm{ }} - {\mathrm{ }}\left( {\nu _{{\mathrm{tip}}}} \right)^2} \right)/E_{{\mathrm{tip}}} + \left( {1{\mathrm{ }} - {\mathrm{ }}\left( {\nu _{{\mathrm{sample}}}} \right)^2} \right)/E_{{\mathrm{sample}}}$$

where *E*_tip_, *E*_sample_ and *ν*_tip,_ *ν*_sample_ are Young’s modulus and Poisson’s ratio of the indentation tip and sample, respectively. Young’s modulus and Poisson’s ratio of the tip were 200 GPa and 0.27, respectively. Poisson’s ratio of the hydrogel and tissue sample were both assumed to be 0.45^63^.

### Preparation of the 3D-aligned cell construct

A double-sided polyimide tape (3M, Maplewood, MN, USA) was used to immobilize the superstrate on the piezoelectric substrate and to create a space for the coupling liquid between the superstrate and the substrate. Two pieces of polyimide tape were attached outside of the SSAW working area to eliminate any interaction with SSAW. The detachable superstrate was placed on the spacers, and coupling liquid was injected into the gap between the SSAW device and the superstrate. Cesium chloride (Samchun Pure Chemical Co., Pyeongtaek, Gyeonggi-do, Korea) solution with a density of 1.9 g cm^−^^3^ was used as a coupling liquid.

hADSCs (Invitrogen, Carlsbad, CA, USA) were cultured in MesenPRO RS basal medium (Invitrogen) supplemented with MesenPRO RS growth supplement (Invitrogen), 1% penicillin/streptomycin (Invitrogen), and 1% GlutaMAX (Invitrogen). HUVECs (Lonza, Basel, Basel-Stadt, Switzerland) were cultured in endothelial cell growth medium-2 (EGM-2).

To prepare the 3D-aligned cell constructs, HUVECs and hADSCs were mixed at ratios of 1:0, 5:1, and 2:1 at a final density of 1 × 10^7^ cells ml^−1^ for in vitro study and 1.2 × 10^7^ cells ml^−1^ for in vivo study. The cell mixtures were suspended into the HA-CA pre-gel solution, and NaIO_4_ solution was added to the cell-laden pre-gel solution for gelation. Immediately after inducing gelation, the mixtures were loaded into the PDMS chamber on the SSAW device.

The piezoelectric substrate was actuated by the sinusoidal electrical signal at a frequency of 13.928 MHz. The SSAW formed by the superposition of two waves with the same magnitude yet opposite directions produced the standing pressure field inside the hydrogel, leading to the patterning of cells. The SSAW was applied for 10 s. The temperature of hydrogel containing cells was maintained below 36 °C using the cooling system during experiment.

After complete gelation, the cell-laden hydrogel construct with the PDMS chamber was transferred to a culture dish and cultured in a mixture of EGM-2 and fully supplemented MesenPRO RS medium at a ratio of 2:1 for 1 week. For analysis of VEGF secretion, the construct was cultured in VEGF-free medium.

### Preparation of hiPSC-EC

The undifferentiated hiPSCs (BJ1) were maintained in mTeSR^TM^ 1 (STEMCELL Technologies, Vancouver, BC, Canada) on 5% matrigel at 37 °C, 5% CO_2_. Differentiation of hiPSCs into ECs was performed following a protocol published by Lee et al.^64^ Briefly, for mesodermal differentiation, enzymatically dissociated clumps of hiPSCs (lower than passage 60) were cultured on 0.01% collagen-coated plates in Dulbecco’s Modified Eagle’s Medium/Nutrient Mixture F-12 (DMEM/F12) medium (Gibco BRL, Gaithersburg, MD, USA) including 20% Serum Replacement (SR, Gibco BRL) with CHIR99021 (3 μM) (STEMCELL Technologies) and fibroblast growth factor 2 (FGF2, 5 ng ml^−1^) (R&D systems, Minneapolis, MN, USA) for 3 days. The mesodermally differentiated cells were cultured in DMEM/F12 medium including 20% SR with VEGF-A (10 ng ml^−1^) (R&D systems), delta-like ligand 4 (DLL4, 25 ng ml^−1^) (R&D systems), epidermal growth factor (EGF, 5 ng ml^−^^1^) (R&D systems), and FGF2 (5 ng ml^−1^) for another 11 days, and hiPSC-derived CDH5 + cells were purified by magnetic-activated cell sorting (MACS, Miltenyi Biotec, Auburn, CA, USA)^64^.

### Viability test

Cytotoxicity was evaluated by staining cells with a live/dead solution (Invitrogen) according to the manufacturer’s instructions. Cells stained with green fluorescence for live cells and red fluorescence for dead cells were observed using a confocal microscope (LSM 880, Carl Zeiss, Oberkochen, Baden-Württemberg, Germany). The percentage of viable cells was calculated by counting the live and dead cells in the confocal images.

### Immunocytochemistry

Cell-laden hydrogel constructs were fixed with 2% formaldehyde (Sigma) for 1 h and then permeabilized with 0.2% Triton X-100 (Wako, Osaka, Japan) for 30 min at room temperature. To prevent nonspecific antibody binding, the constructs were incubated with 5% bovine serum albumin (Wako) for 2 h at room temperature. The following primary antibodies were applied for 48 h at 4 °C: mouse anti-CD31 (Millipore, Schwalbach, Hessen, Germany, Cat# CBL468, 1:20 dilution), rabbit anti-CD44 (Proteintech, Chicago, IL, USA, Cat# 15675-1-AP, 1:100 dilution), rabbit anti-VE-cadherin (Cell Signaling Technology, Beverly, MA, USA, Cat# 2500S, 1:100), mouse anti-α-SMA (Chemicon, Temecula, CA, USA, Cat# CBL171, 1:1000 dilution), and rabbit anti-ZO-1 (Invitrogen, Cat# 61-7300, 1:50 dilution). The constructs were then washed with PBS overnight at 4 °C and incubated with the following secondary antibodies overnight at 4 °C: Alexa Fluor 488-conjugated goat anti-mouse IgG (Invitrogen, Cat# A11001, 1:200 dilution) and Alexa Fluor 594-conjugated goat anti-rabbit IgG (Invitrogen, Cat# A11002, 1:200 dilution). Nuclei were identified by staining with 4ʹ,6-diamidino-2-phenylindole (DAPI; Vector Laboratories, Burlingame, CA, USA) for 30 min at room temperature. A confocal microscope (LSM 880) was used to observe the stained cells.

### Image-based quantification

To visualize the spatial distribution of cells in the hydrogel, DiI (Invitrogen)-stained HUVECs or green fluorescent protein (GFP)-overexpressing HeLa cells were patterned in 2% HA-CA hydrogel. The cell density was varied from 1 × 10^6^ cells ml^−1^ to 2 × 10^7^ cells ml^−1^. For time-lapse imaging, DiI-stained cells at a density of 1 × 10^7^ cells ml^−1^ were patterned in polymerizing 2% HA-CA hydrogel. Time-lapse images of cells during patterning were obtained by an epifluorescence microscope (Ni-U, Nikon, Tokyo, Japan) with a charge-coupled device (CCD) camera (DS-Q1Mc, Nikon). The blood vessels in mouse hindlimb muscle tissue were visualized via the tail vein injection of 70 kDa FITC-dextran (Sigma) solution. Fluorescence signals of GFP-overexpressing HeLa cells and FITC-dextran in blood vessels were imaged using a confocal microscope (LSM 880).

The alignment index (AI) of the blood vessels in tissue samples and the patterned cells in hydrogel samples were obtained by the 2D fast Fourier transform (FFT) approach^65^ using Fiji software^66^ (National Institutes of Health, Bethesda, MD, USA) (Supplementary Fig. 1). The 2D Fourier transforms of fluorescent images of tissues and cells were obtained, and the summation of pixel intensities along a line from the center of the 2D FFT images at an angle from 0° to 180° was measured using Fiji software (National Institutes of Health). The peak of the intensity was recentered to 0°, and the ratio of the area of the normalized intensity within 20° around the peak to the whole area of the intensity was calculated. The AI of an image was calculated by dividing the area ratio of the image by the ratio of an image with randomly distributed objects (40/180 = 0.22). Higher AI values indicate that the objects in the image are more aligned. The AI value varied from 1 to 4.5.

The average intensity projections (AIP) of the images of patterned cells were acquired using Fiji software (National Institutes of Health). The intensity profiles of cells along the *X*, *Y*, and *Z* axes were obtained from the projected images and then normalized to the maximum value of the intensity profile.

To characterize elongated ECs in a quantitative manner, a projection image of 3D confocal images of CD31-stained cells with 40-μm thickness was obtained. Directionality of the ECs was estimated using the ‘Directionality’ function in Fiji software (National Institutes of Health) for the stacked images. The length of the vessel branch was estimated by converting a stacked image into a skeletonized image and using the ‘Analyze Skeleton (2D/3D)’ function.

To assess the physical contact between cells, aggregated cells in 3D confocal images were rendered into a single object using the ‘Surface’ function in Imaris software (Bitplane, Zurich, Switzerland). The volume of the separated objects was visualized by the ‘Statistics’ function in Imaris MeasurementPro software (Bitplane).

### Analysis of paracrine factor secretion

Conditioned medium was collected from each culture dish with cell-laden hydrogel constructs at days 1, 3, 5, and 7. Paracrine factors, including human VEGF, human IL-10 and human PDGF in the collected conditioned medium were detected by enzyme-linked immunosorbent assay (ELISA) kits (R&D Systems) following the manufacturer’s instructions.

### Quantitative real-time polymerase chain reaction (qPCR)

To isolate cells from the HA-CA hydrogels, the cell-laden hydrogel constructs were incubated in 1000 U ml^−^^1^ of hyaluronidase solution for 20 min at 37 °C. Degraded hydrogel solution was then centrifuged at 1000 rpm for 5 min to collect the cell pellet. After decanting the supernatant, the cell pellet was lysed, and total RNA was extracted using a MiniBEST universal RNA extraction kit (TaKaRa, Otsu, Shiga, Japan). RNA samples were reverse-transcribed into cDNA using a PrimeScript^TM^ strand cDNA synthesis kit (TaKaRa). qPCR was conducted with the synthesized cDNA using a StepOnePlus Real-Time PCR system (Applied Biosystems, Foster City, CA, USA), TaqMan Fast Universal PCR Master Mix (Applied Biosystems), and TaqMan gene expression assays (VE-cadherin (*CDH5*): Hs00901465_m1, α-SMA (*ACTA2*): Hs00426835_g1, Tie2 (*TEK*): Hs00945150_m1 and vWF (*VWF*): Hs01109446_m1). Target gene expression value was determined by the comparative *C*_t_ method and normalized to that of an endogenous housekeeping gene, glyceraldehyde 3-phosphate dehydrogenase (*GAPDH*: Hs02758991_g1).

### Subcutaneous transplantation of the constructs

All animal experiments in the study were reviewed and approved by the Institutional Animal Care and Use Committee (IACUC) at the Yonsei Laboratory Animal Research Center (YLARC; permit number: IACUC-A-201603-172-02). Immediately after fabricating the cell-hydrogel constructs in vitro, the constructs were transplanted into subcutaneous pockets in the dorsal regions (two constructs per mouse; right side for the ‘Align’ group and left side for the ‘Random’ group) of 6-week-old female Balb/c-nu mice (Orient Bio, Seongnam, Gyeonggi-do, Korea). After a longitudinal incision was made in the mouse dorsal skin, each construct was placed between the skin and muscle layer. The incision was closed with a 6-0 prolene suture (Ethicon, Somerville, NJ, USA). To visualize the vasculatures of the transplanted constructs and the surrounding skin tissue at 1 and 3 weeks post-transplantation, 100 μl of 1 mg ml^−^^1^ FITC-dextran (70 kDa, Sigma) or fluorescein-isolectin solution (Vector Laboratories) was intravenously injected into the mouse tail vein. The mice were sacrificed 15 min after injection, and the hydrogel constructs were harvested while attached to skin tissue. The fluorescently labeled vasculatures in the harvested constructs were immediately visualized using a confocal microscope (LSM 880). Constructs with skin tissue were also fixed with 4% paraformaldehyde (Sigma) overnight at room temperature and embedded in optimal cutting temperature (OCT) compound (CellPath, Newtown, Powys, UK) for histological analysis. After cryosectioning, the slices were stained with H&E and immunofluorescently stained for CD31 (Abcam, Cambridge, Cambridgeshire, UK) to confirm the capillaries in the tissue constructs following standard histological procedures.

### Transplantation of the constructs in hindlimb ischemia model

Hindlimb ischemia was induced in anesthetized athymic female mice (Balb/c-nu, 6 weeks old, Orient Bio)^49^. After skin incision, the left iliac and femoral arteries were permanently ligated using a 6-0 prolene suture (Ethicon). Immediately after ligation of the arteries, 3D cell-hydrogel constructs, pre-matured in vitro for 1 week, were placed onto the defective muscle, fully covering the ischemic region. The mice were divided into four groups: (i) no-treatment, (ii) hydrogel-alone (Gel), (iii) randomly distributed cell-hydrogel construct (Random), and (iv) aligned cell-hydrogel construct (Align). The status of the lower extremity of the left limb on day 28 was evaluated to determine its physiological status score. Amputation of the lower extremity was designated as limb loss, and rotten skin and muscle of the left limb was designated as necrosis. The blood perfusion in ischemic hindlimbs was monitored by serial scanning using a laser Doppler imaging system (MoorLDI2-HIR High Resolution, Moor Instruments, Axminster, Devon, UK) on days 0, 2, 7, 14, 21, and 28 after treatment^67^. Blood flow was quantified using digital color-coded images from the knee joint region to the toe region, and the perfusion rate was calculated as the ratio of ischemic limb to normal limb. Four weeks after transplantation, the mice were sacrificed for histological analysis.

### Histology and immunohistochemistry

Harvested ischemic muscles were fixed with 4% paraformaldehyde (Sigma) overnight at room temperature and embedded in paraffin for slicing. The tissue slices were stained with H&E and Masson’s trichrome. The fibrotic area was quantified as the percentage of collagen-stained area in Masson’s trichrome-stained images using Fiji software (National Institutes of Health). The sectioned specimens were immunofluorescently stained for capillaries and arterioles using anti-CD31 (Abcam, Cat# ab9498, 1:50 dilution) and anti-α-SMA antibodies (Santa Cruz Biotechnology, Santa Cruz, CA, USA, Cat# sc-53142, 1:100 dilution) following standard histological procedures. The densities of capillaries and arterioles were determined by counting CD31-positive capillaries and α-SMA-positive microvessels, respectively, and arteriole size was measured using Fiji software (National Institutes of Health) based on the fluorescent images of α-SMA-positive microvessels. The sectioned tissues were also immunofluorescently stained to detect blood vessels formed by transplanted human cells using human-specific anti-CD44 (Abcam, Cat# ab51037, 1:100 dilution) and anti-CD31 (Abcam, Cat# ab9498, 1:50 dilution). The number of blood vessels consisting of transplanted hADSCs was determined by counting the human CD44-positive vessels in the field of view.

### Statistical analysis

All data are presented as the mean ± standard deviation and were statistically analyzed using GraphPad Prism (GraphPad Software, San Diego, CA, USA). To determine statistical significance, unpaired Student’s *t*-tests and one-way or two-way analysis of variance (ANOVA) were used.

## Supplementary information

Supplementary Information

## Data Availability

The authors declare that all data supporting the findings of this study are available within the paper and its supplementary information file.
