# Supplementary material for: High-resolution acoustophoretic 3D cell patterning to construct functional collateral cylindroids for ischemia therapy
Source: Nat Commun. 2018 Dec 20;9:5402. doi: 10.1038/s41467-018-07823-5 (PMC6302096; doi:10.1038/s41467-018-07823-5)
Supplement: Supplementary file 1 — Supplementary Information [file 41467_2018_7823_MOESM1_ESM.pdf]

Supplementary information

High-resolution acoustophoretic 3D cell patterning to construct functional collateral cylindroids for ischemia therapy

Kang et al.

Supplementary Figures

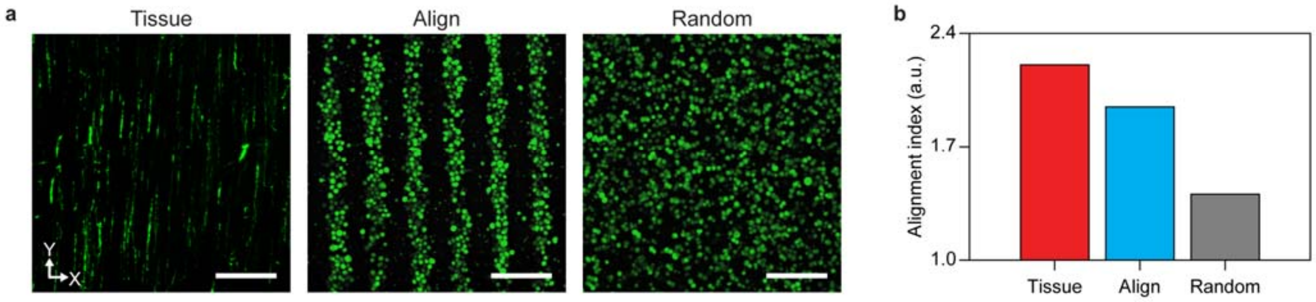

Supplementary Figure 1. Image-based analysis of alignment index. (a) Projection images of 70 kDa FITC-dextran in blood vessels in hindlimb tissue (Tissue), aligned GFP-overexpressing HeLa cells in 2% HA-CA gel (Align), and randomly distributed GFP-overexpressing HeLa cells in 2% HA-CA gel (Random). Scale bars = 200 μm. (b) Alignment index obtained from 2D-FFT analysis for those fluorescent images.

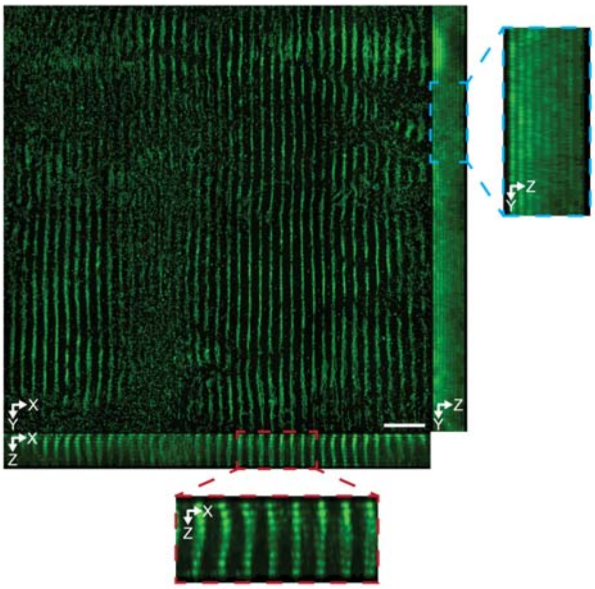

Supplementary Figure 2. XY/XZ/YZ AIP images of GFP-overexpressing HeLa cells in 2% HA-CA hydrogel with a dimension of 6 × 6 × 0.53 mm at the macroscale. The inset shows a zoom-in of the image within the box in a dotted line. Scale bar = 500 μm.

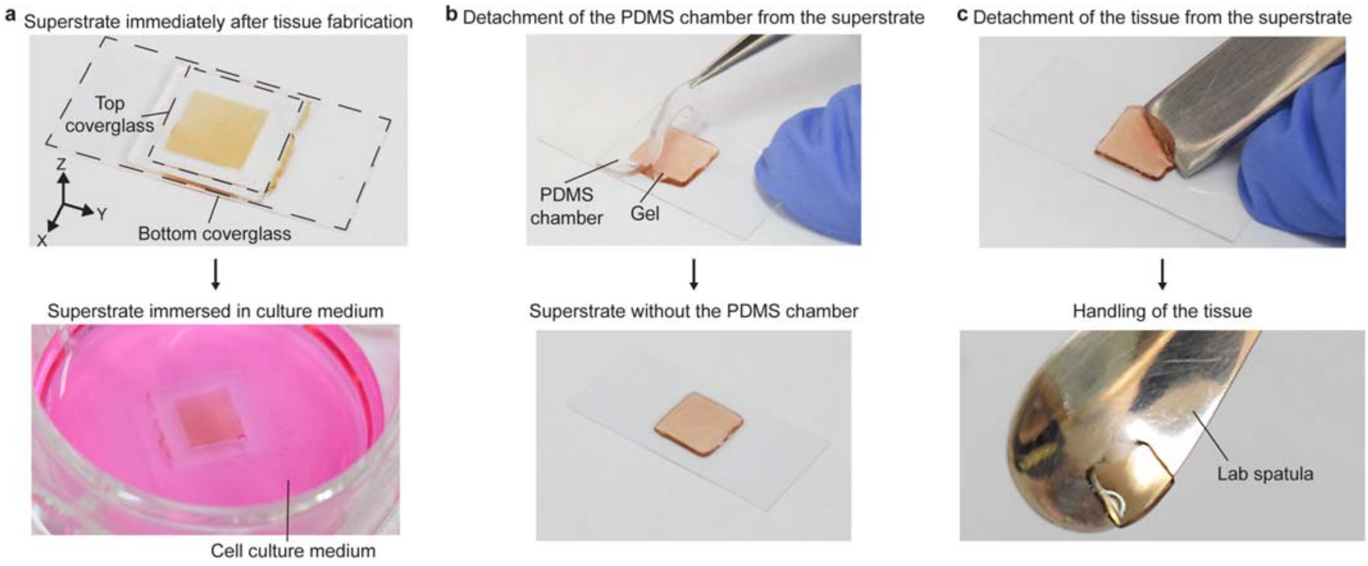

Supplementary Figure 3. Separation of the fabricated tissue from the superstrate. (a) After removing the top coverglass, the superstrate was immersed in culture medium. (b) The PDMS chamber was detached from the superstrate. (c) The cell-gel tissue construct was detached from the bottom coverglass using a Lab spatula.

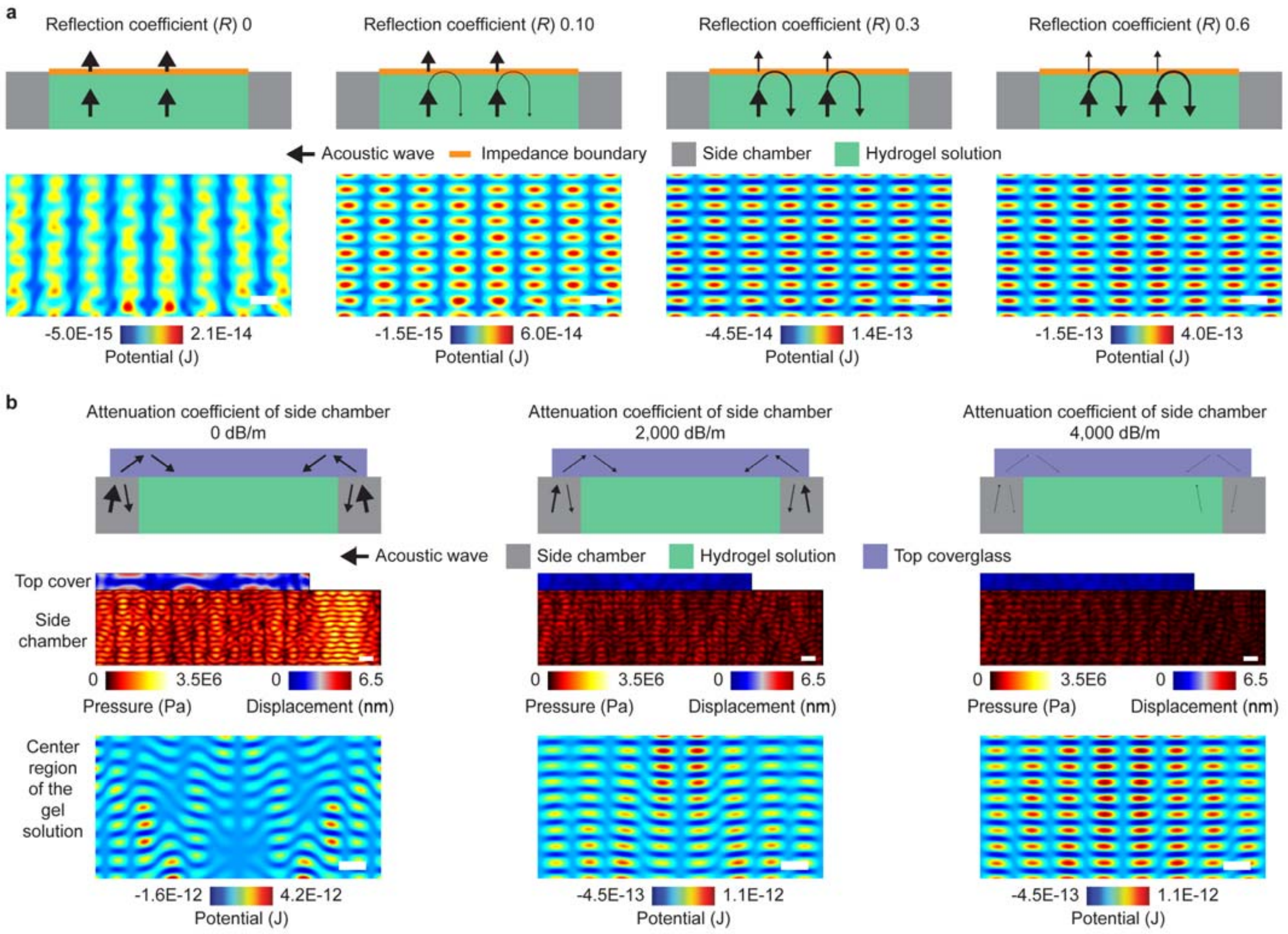

Supplementary Figure 4. Effects of the cover and side chamber of our device on the acoustic pressure field in the gel solution. (a) Effect of the wave reflection at the interface of gel solution/top cover interface on the acoustic field inside the gel solution. A schematic of the wave reflection and the acoustic potential field in the center region of the solution as a function of the reflection coefficient *R*, which was defined as follows[1]: $R = ((Z_{cover}-Z_{fluid})*(Z_{cover}+Z_{fluid})^{-1})^2$ where Z_{cover} and Z_{fluid} are the acoustic impedance of the cover and the fluid, respectively. Scale bars = 100 μm. As the reflection coefficient increased, the clearer pattern of the potential field was exhibited along Z direction. (b) Effect of wave attenuation in the side chamber on the acoustic field of the gel solution. (Top row) Schematics to show wave transmission from the side chamber to the coverglass. (Middle row) Time-averaged pressure fields in the side chamber and displacement at the top cover. (Bottom row) Acoustic potential field in the center region of the gel solution. Scale bars = 100 μm.

When the attenuation coefficient of side chamber was small, the wave from the side chamber readily propagated into the coverglass leading to the relatively large displacement. Due to the wave reflection at the top cover/air interface, the wave can be transmitted into the gel solution disturbing the acoustic field in the gel solution. As the attenuation coefficient of side chamber increased, the effect of the wave propagated from the side chamber on the acoustic field in the gel solution was reduced. As a result, the clearer patterns in the potential fields along the both X and Z directions were exhibited.

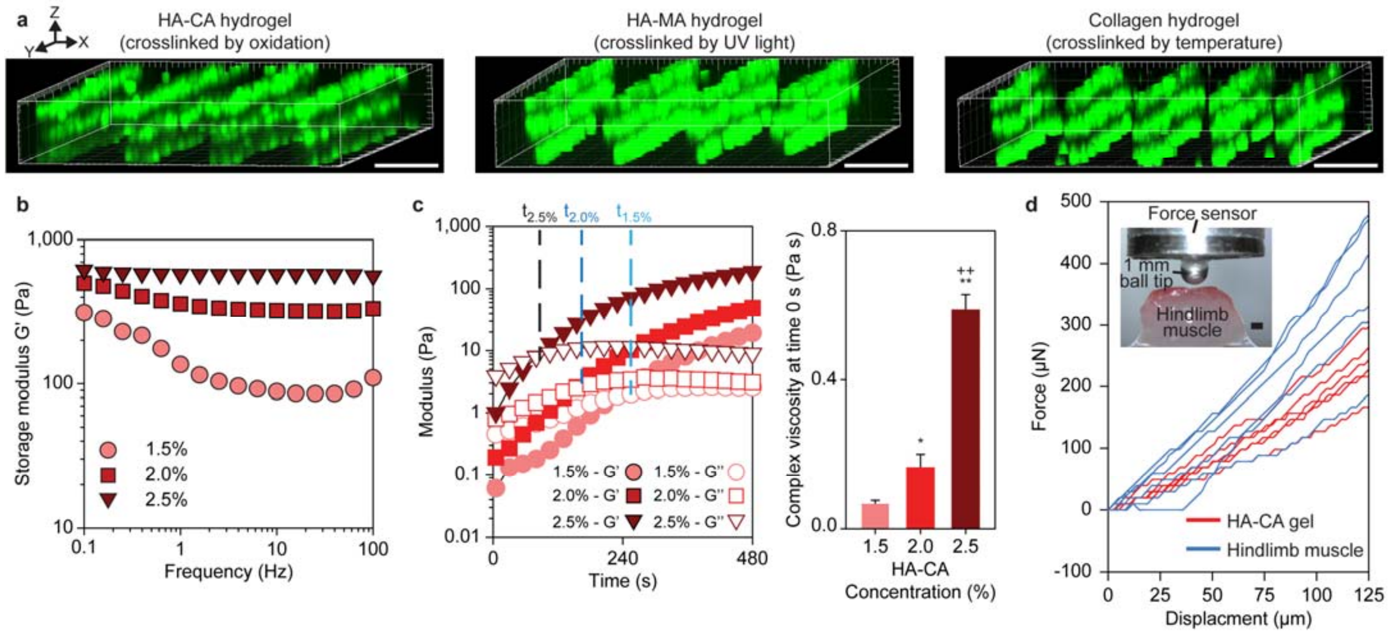

Supplementary Figure 5. Application of the 3D cell patterning to various hydrogels (a) and mechanical properties of the HA-CA hydrogel (b-d). (a) 3D images of GFP-overexpressing HeLa cells patterned in HA-CA hydrogel (crosslinked by oxidation), HA-MA hydrogel (crosslinked by UV light), and collagen hydrogel (crosslinked by temperature). Scale bars = 100 μm. The similar pattern of cells was observed independent of type of hydrogel. (b) The storage modulus G' of the HA-CA hydrogel measured in frequency sweep rheology tests. (c) Time-dependent storage (G') and loss (G'') modulus of HA-CA hydrogel during gelation at various concentrations, and the complex viscosity of HA-CA pre-gel solution at the initiation of gelation (t = 0 s, n = 4, *$p < 0.05$ and **$p < 0.01$ versus 1.5% HA-CA group, ++$p < 0.01$ versus 2.0% HA-CA group). The gelation time ($t_{1.5\%}$, $t_{2.0\%}$, $t_{2.5\%}$) was defined as the time at which the storage modulus and the loss modulus intersected[2]. The complex viscosity was defined as $\mu_{complex} = ((G'\omega^{-1})^2+(G''\omega^{-1})^2)^{0.5}$, where ω is the angular frequency of the oscillatory rheology[3]. Error bars represent one standard deviation. (d) Force-displacement curves of 2% HA-CA gel and mouse hindlimb muscle obtained from indentation tests using a 1-mm ball tip. The inset is an image of the indentation experiment for mouse hindlimb muscle tissue. Scale bar = 0.5 mm. The 2% HA-CA hydrogel and hindlimb muscle showed the similar mechanical behaviors in response to indentation.

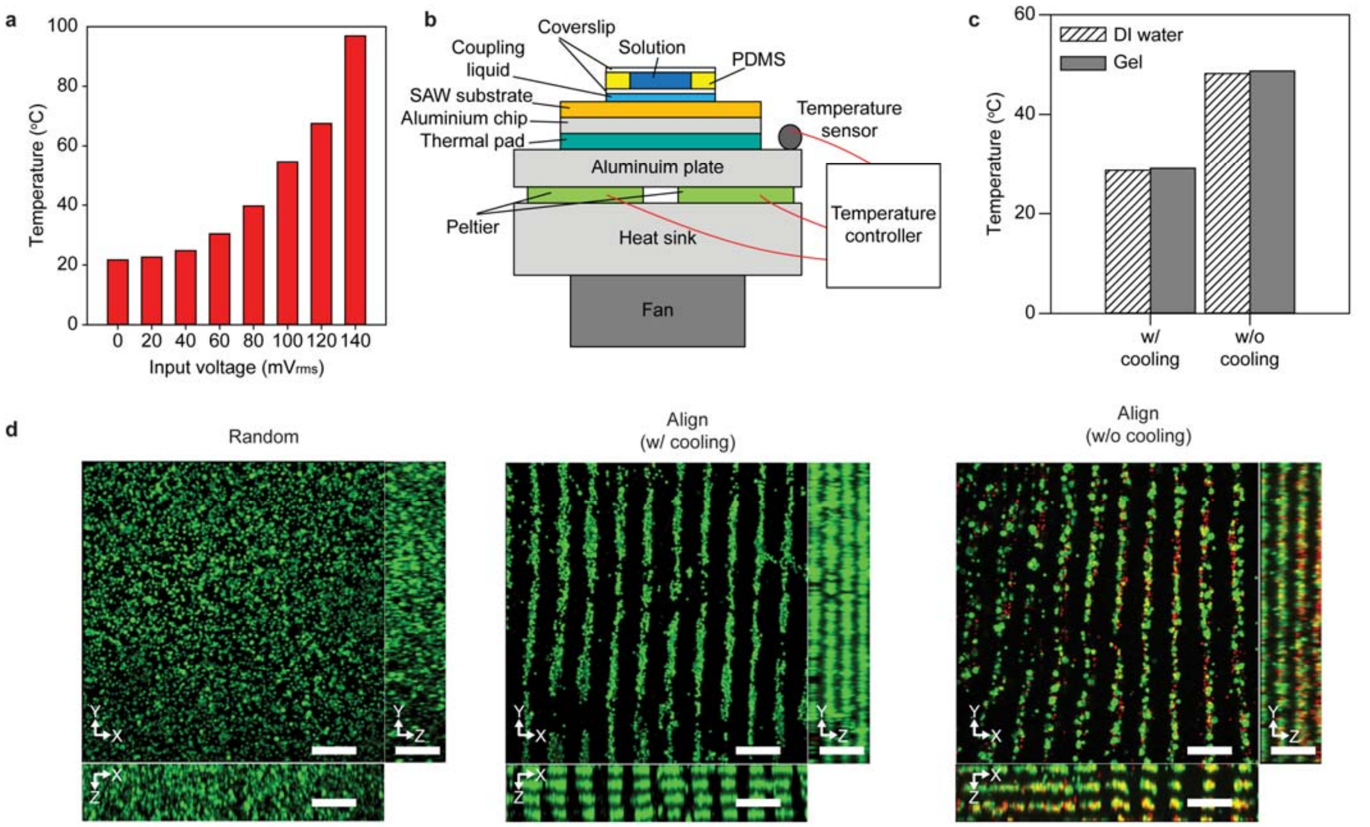

Supplementary Figure 6. Temperature control using the Peltier device. (a) Steady-state temperature of water subject to SSAW without the cooling system. (b) Schematic of the SSAW device (SSAW substrate, coupling liquid, coverslip, PDMS, aluminium chip, and solution) equipped with the cooling system (aluminum plate, Peltier device, heat sink, fan, temperature sensor, and temperature controller). (c) Steady-state temperature of water and 2% HA-CA hydrogel solution at an input voltage of 90 mVrms with and without the cooling system. (d) Live/dead images of the Random, Align (w/ cooling) and Align (w/o cooling) groups. Red and green in images represent dead and living cells, respectively. Scale bars = 200 µm.

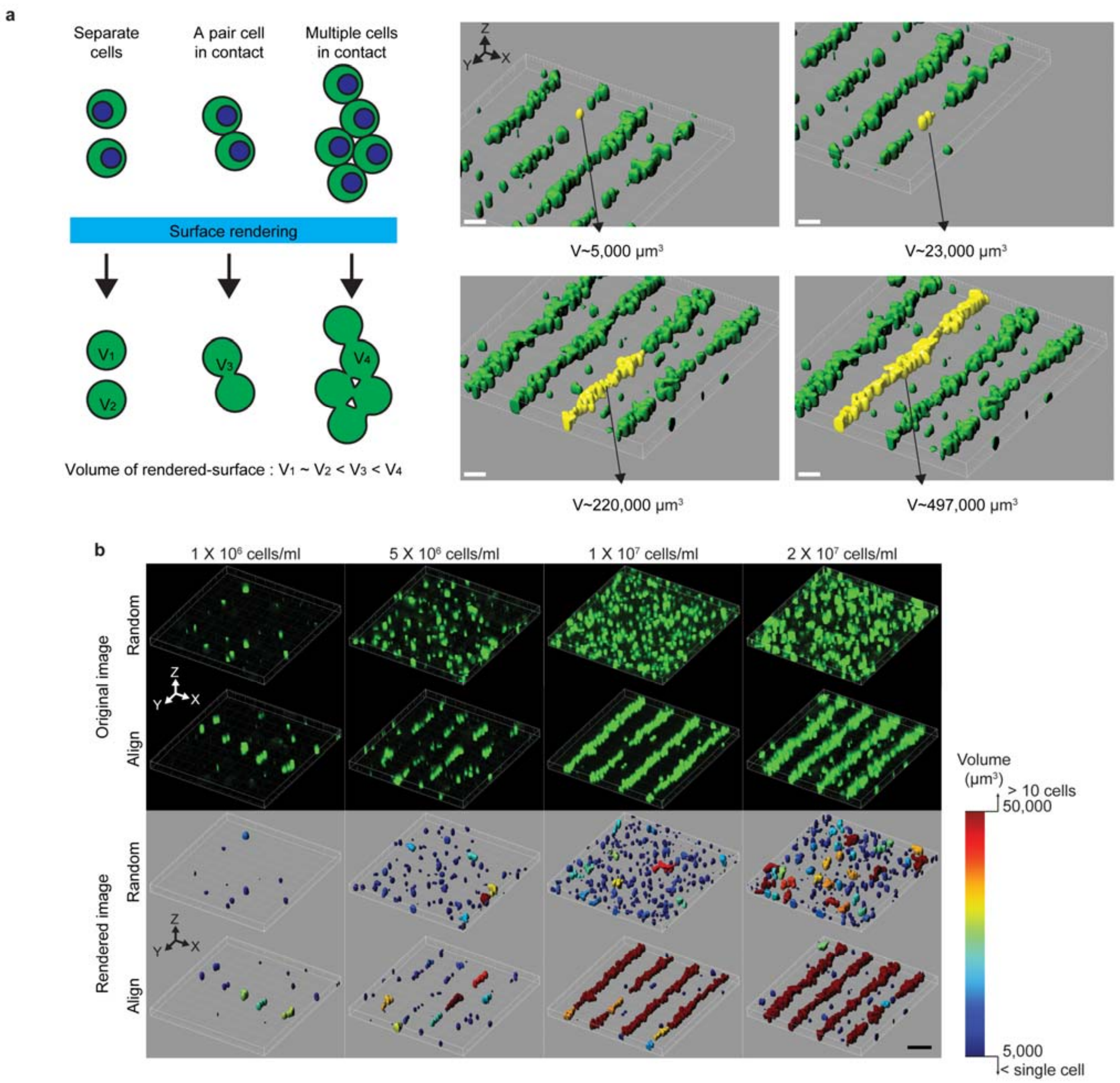

Supplementary Figure 7. Estimating volume of cells in contact. (a) Estimating volume of single cells and cells in contact using surface rendering. Scale bars = 50 μm. A volume of single cell was estimated to be 5000 μm^2 approximately. (b) Original and surface-rendered 3D confocal images of randomly distributed cells (Random) and aligned cells (Align) at various cell densities. Scale bar = 100 μm. The color bar indicates the volumes of rendered surfaces of contacting cells.

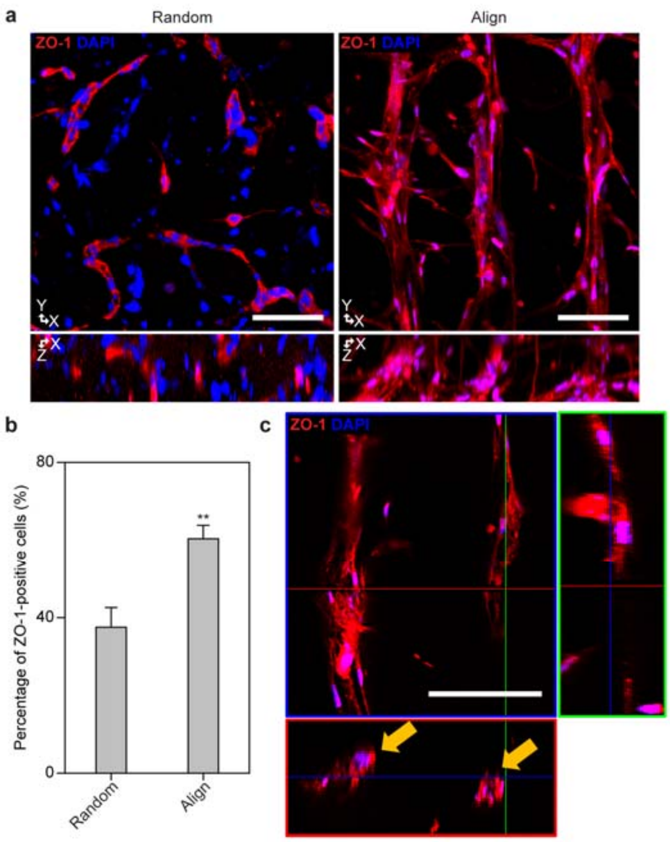

Supplementary Figure 8. Formation of tight junction in a 3D aligned vascular cell construct. (a) Fluorescent images showing the expression of the epithelial tight junction marker ZO-1 at 2:1 HUVEC/hADSC ratio (total cell density: 1×10^7 cells ml^{-1}) 1 week after cultivation. Scale bars = 100 µm. (b) Quantification of ZO-1-positive ECs in a random and aligned 3D cell-hydrogel construct ($n = 3$-4, $^{**}p <$ 0.01 versus Random group). Error bars represent one standard deviation. (c) Magnified confocal ortho-view images of 3D aligned vascular cell construct at 2:1 ratio of HUVEC/hADSC (total cell density: 1×10^7 cells ml^{-1}) 1 week after cultivation. Yellow arrows indicate vascular lumen-like structures within the 3D-aligned cell construct. Scale bar = 100 µm.

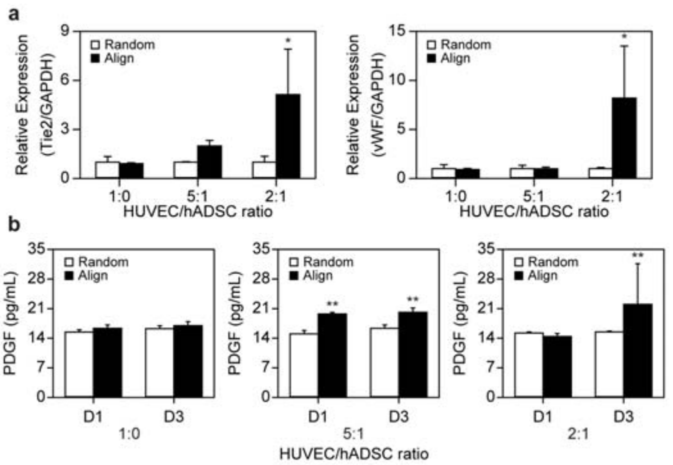

Supplementary Figure 9. Functional maturation of 3D aligned vascular construct. (a) qPCR analysis to quantify the expression of EC-specific genes Tie2 (left) and vWF (right) in aligned/randomly distributed cells within the hydrogel at 7 days in culture (n = 3-4, *$p < 0.05$ versus each random group). (b) ELISA analysis of PDGF secretion by aligned/randomly distributed cells in hydrogels at day 1 and 3 (n = 3, **$p < 0.01$ versus Random group at each time point). Error bars represent one standard deviation.

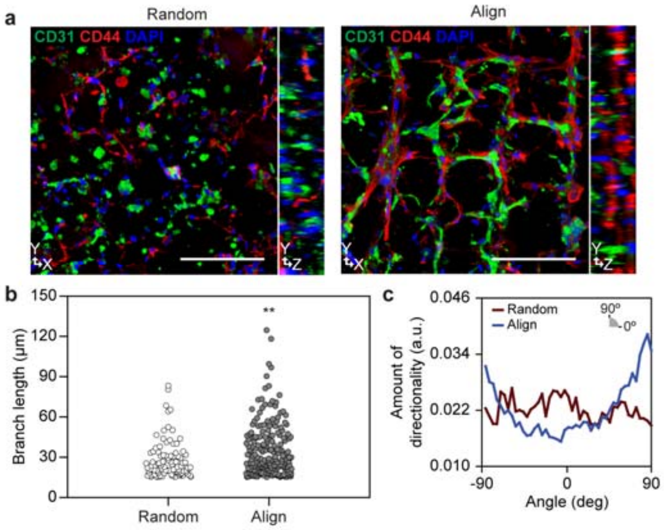

Supplementary Figure 10. SSAW-induced 3D cell patterning with hiPSC-ECs and hADSCs. Dual immunofluorescence staining of CD31 (green) for iPSC-ECs and CD44 (red) for hADSCs in (a) randomly distributed (left) and aligned (right) 3D construct at 2:1 hiPSC-EC/hADSC ratio (total cell density: 1×10^7 cells ml^{-1}) 3 days after cultivation. Scale bars = 200 µm. Fluorescent image-based quantification of (b) hiPSC-EC elongation (n =97-214) and (c) elongated direction using CD31-stained images (**p < 0.01 versus Random group).

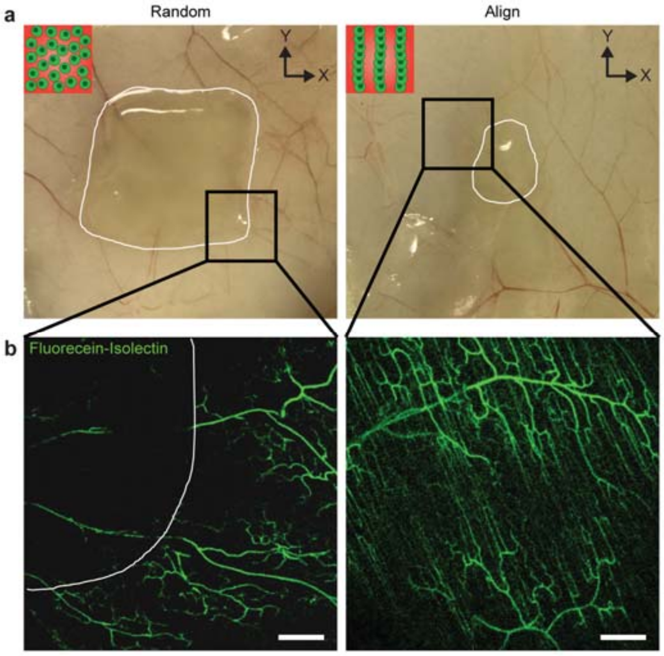

Supplementary Figure 11. Three-week observation of subcutaneously transplanted 3D-aligned cell-hydrogel construct. (a) Gross views of harvested back-skin tissues with a cell-hydrogel construct 3 weeks after transplantation. (b) Fluorescent images of vessels in a 3D cell-hydrogel construct and adjacent skin tissue immediately after the tail vein injection of fluorescein-isolectin 3 weeks post-transplantation. Scale bars = 200 μm.

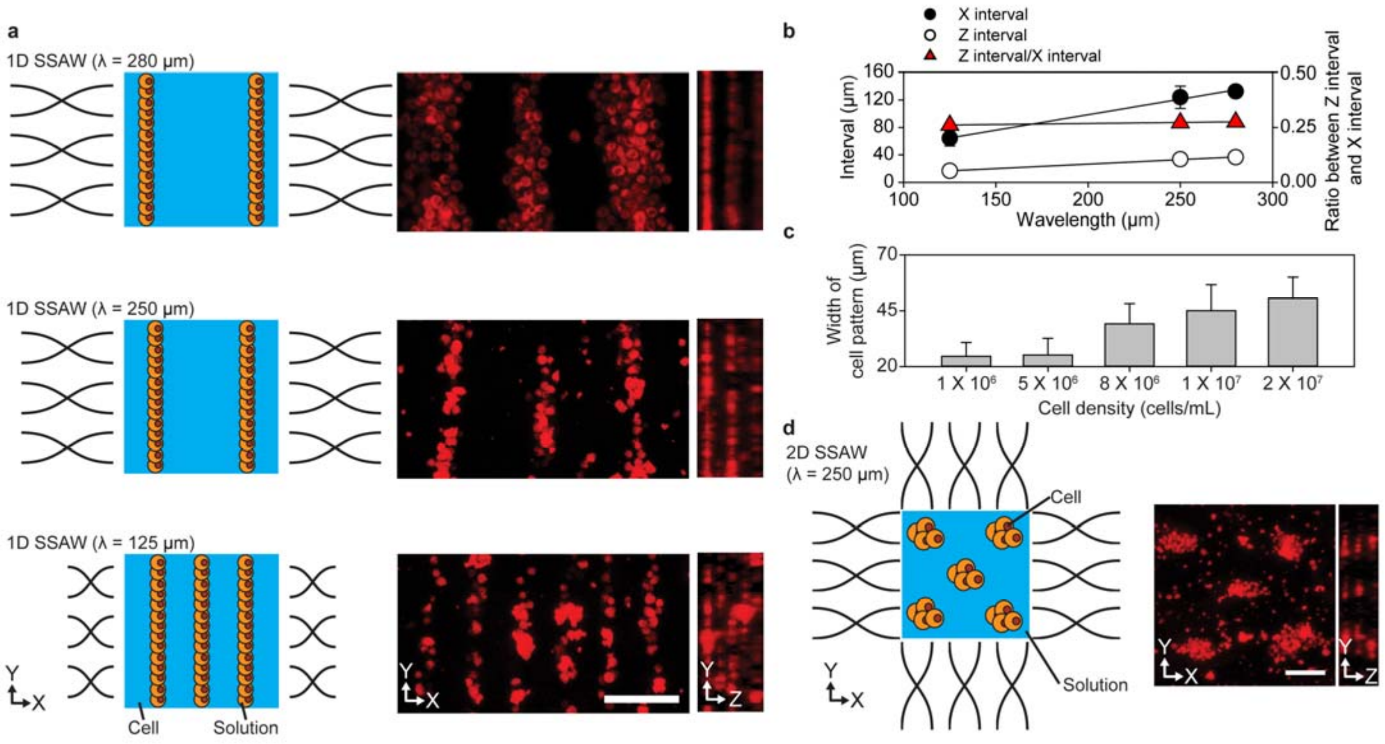

Supplementary Figure 12. Various patterns of cells and microparticles. (a) Line patterns of cells with various spacings. Fluorescent images of DiI-stained cells patterned in a hydrogel using 1D SSAW with various wavelengths of 280 μm, 250 μm, and 125 μm. Scale bar = 100 μm. (b) X- and Z-directional interval as a function of SSAW wavelength (n = 8-43). (c) Width of the aligned cell patterns by 280 μm wavelength SAW as a function of cell density (n = 5-16). (d) Dot-array pattern of cells. Fluorescent images of DiI-stained cells patterned in a hydrogel using 2D orthogonal SSAWs. Scale bar = 100 μm. Error bars represent one standard deviation.

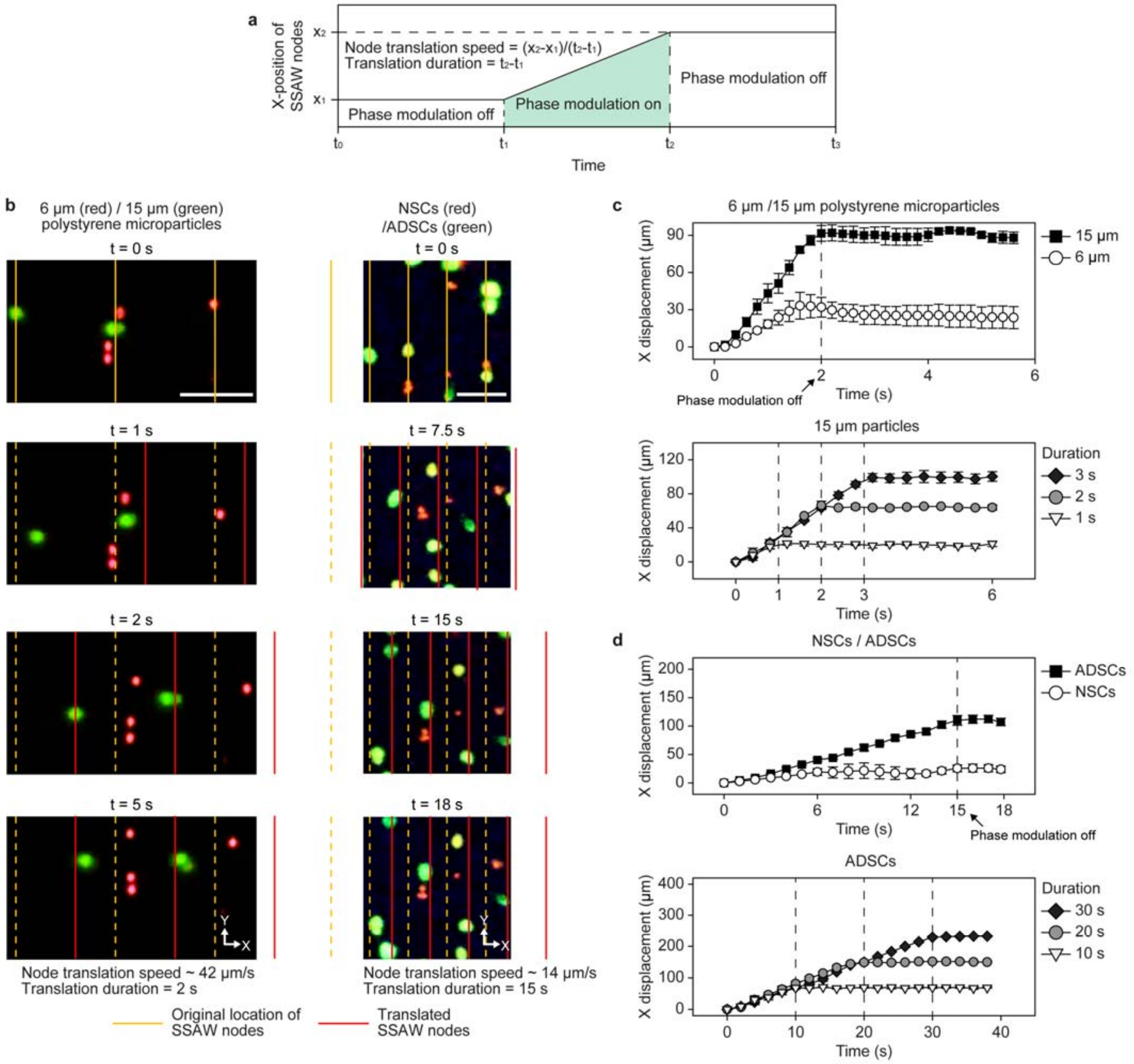

Supplementary Figure 13. Selective positioning of particles and cells using SSAW with the phase modulation technique. (a) X-position of SSAW nodes changed by modulating a phase of one of the counter-propagating waves in SSAW. (b) Fluorescent time-lapse images of 6 μm/15 μm polystyrene microparticles and neural stem cells (NSCs)/adipose-derived stem cells (ADSCs) in subject to the phase-modulated SSAW. Scale bars = 100 μm. (c) X-direction displacements of 6 and 15 μm microparticles in response to the phase-modulated SSAW. The phase-modulated SSAW is on for the first 2 s and then turned off (n = 3) (Top). Displacements of 15 μm particle at varied durations of phase-modulated SSAW application (n = 3) (Bottom).

(d) X-directional displacements of NSCs and ADSCs in response to the phase-modulated SSAW. The phase-modulated SSAW is on for the first 15 s and then turned off (n = 3) (Top). Displacements of ADSCs at varied durations of the phase-modulated SSAW application (n = 3) (Bottom). Speeds of the translating nodes were ~ 42 μm s^{-1} and ~ 14 μm s^{-1} in experiments for particles and cells, respectively. Error bars represent one standard deviation.

Supplementary Tables

Material	Property	Value	Unit
PDMS	Density[4]	965	kg m^{-3}
	Speed of sound[4]	1020	m s^{-1}
	Attenuation coefficient (calculated by fitting the experimental data from a previous study[5] to the power law[6]	4340.61	dB m^{-1}
Cell	Density[7,8]	1060	kg m^{-3}
	Isentropic compressibility[8]	366	TPa^{-1}
	Size	20	µm

Supplementary Table 1. Material properties used in the computational study.

Property	Value	Unit
Frequency	13.928	MHz
SAW velocity	3900	m s^{-1}
SAW wavelength	280	µm
SAW voltage	50	V

Supplementary Table 2. SAW parameters used in the computational study.

Supplementary References

1. Cobbold, R. S. *Foundations of biomedical ultrasound Ch. 1* (Oxford University Press, Oxford, 2006).
2. Ouyang, L., Yao, R., Zhao, Y. & Sun, W. Effect of bioink properties on printability and cell viability for 3D bioplotting of embryonic stem cells. *Biofabrication* **8**, 035020 (2016).
3. Mezger, T. G. *The Rheology Handbook: For users of rotational and oscillatory rheometers Ch. 8* (Vincentz Network, Hannover, 2006).
4. Shi, J. *et al.* Three-dimensional continuous particle focusing in a microfluidic channel via standing surface acoustic waves (SSAW). *Lab Chip* **11**, 2319-2324 (2011).
5. Tang, X., Toksöz, M. N., Tarif, P. & Wilkens, R. H. A method for measuring acoustic wave attenuation in the laboratory. *J. Acoust. Soc. Am.* **83**, 453-462 (1988).
6. Tsou, J. K., Liu, J., Barakat, A. I. & Insana, M. F. Role of Ultrasonic Shear Rate Estimation Errors in Assessing Inflammatory Response and Vascular Risk. *Ultrasound Med. Biol.* **34**, 963-972 (2008).
7. Bryan, A. K. *et al.* Measuring single cell mass, volume, and density with dual suspended microchannel resonators. *Lab Chip* **14**, 569-576 (2014).
8. Hartono, D. *et al.* On-chip measurements of cell compressibility via acoustic radiation. *Lab Chip* **11**, 4072-4080 (2011).
